# Systematic discovery of germline cancer predisposition genes through the identification of somatic second hits

**DOI:** 10.1038/s41467-018-04900-7

**Published:** 2018-07-04

**Authors:** Solip Park, Fran Supek, Ben Lehner

**Affiliations:** 1 Systems Biology Program, Centre for Genomic Regulation (CRG), The Barcelona Institute of Science and Technology, Dr Aiguader 88, 08003 Barcelona, Spain; 2 Institut de Recerca Biomedica (IRB Barcelona), The Barcelona Institute of Science and Technology, 08028 Barcelona, Spain; 3 Division of Electronics, Rudjer Boskovic Institute, 10000 Zagreb, Croatia; 4 Universitat Pompeu Fabra (UPF), 08003 Barcelona, Spain; 5 Institució Catalana de Recerca i Estudis Avançats (ICREA), Pg. Luis Companys 23, 08010 Barcelona, Spain; 6 Present Address: Institut de Recerca Biomedica (IRB Barcelona), The Barcelona Institute of Science and Technology, 08028 Barcelona, Spain

## Abstract

The genetic causes of cancer include both somatic mutations and inherited germline variants. Large-scale tumor sequencing has revolutionized the identification of somatic driver alterations but has had limited impact on the identification of cancer predisposition genes (CPGs). Here we present a statistical method, ALFRED, that tests Knudson’s two-hit hypothesis to systematically identify CPGs from cancer genome data. Applied to ~10,000 tumor exomes the approach identifies known and putative CPGs – including the chromatin modifier *NSD1* – that contribute to cancer through a combination of rare germline variants and somatic loss-of-heterozygosity (LOH). Rare germline variants in these genes contribute substantially to cancer risk, including to ~14% of ovarian carcinomas, ~7% of breast tumors, ~4% of uterine corpus endometrial carcinomas, and to a median of 2% of tumors across 17 cancer types.

## Abstract (web summary)

Inherited germline variants and somatic mutations contribute to cancer. Here, the authors present the statistical method ALFRED that tests the two-hit hypothesis of tumorigenesis and apply it to ~10,000 tumor exomes to identify rare germline variants that affect putative cancer predisposition genes, contributing substantially to cancer risk.

## Introduction

Inherited risk for cancer was first proposed by Broca because of the history of breast cancer in 15 members of his wife’s family^1^. However, it was Alfred Knudson’s ‘two-hit’ hypothesis that initiated the identification of cancer predisposition genes (CPGs) in which deleterious germline variants have been associated with increased risks of cancer^2^. Through a statistical analysis of retinoblastoma cases, Knudson proposed that ‘two hits’ to the DNA were necessary to cause cancer and that in children with the inherited form of the disease the first hit is inherited variation in one allele of the gene with the ‘second hit’ being a somatically acquired inactivation of the second allele^3^. This model was confirmed by the identification of biallelic inactivation of the *RB1* gene in retinoblastoma and indeed most known high-penetrance inherited cancer predisposition variants are loss-of-function mutations in recessively acting tumor suppressor (TS) genes^2,4^.

Tumor sequencing has led to the systematic identification of somatically acquired cancer driver alterations^5^. In contrast, to-date, sequencing has had limited success in identifying CPGs^6–9^, with most CPGs having been identified from high-penetrance variants in family studies^2,10^. As for other genetic diseases, an important reason for this is the low statistical power to detect associations between rare genetic variants and disease risk in genome-wide analyses, even in large population studies^11–13^.

We reasoned that Knudson’s original two-hit model provides a more specific hypothesis that can be tested genome-wide to identify CPGs from tumor sequencing data. We present a method to achieve this and its application to the analysis of ~10,000 tumor exomes.

## Results

### ALFRED: discovery of putative cancer predisposition genes

**Fig. 1:**
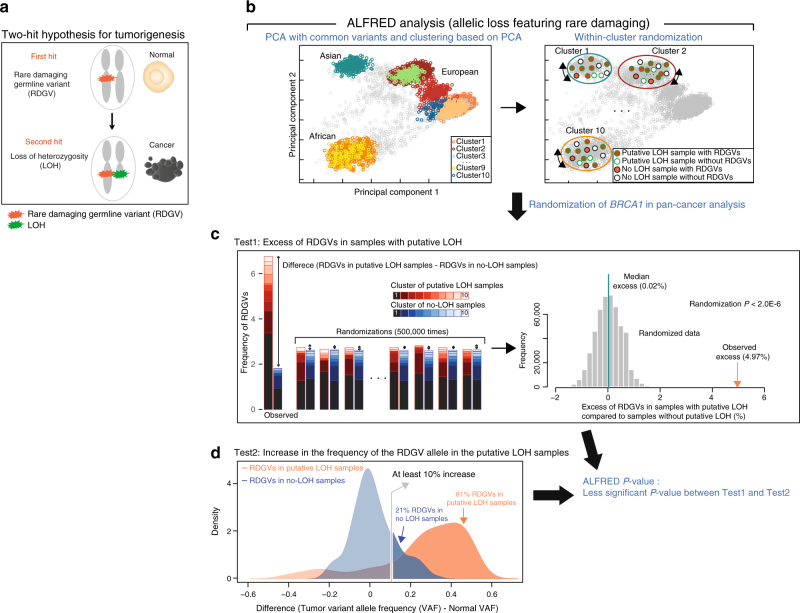
Systematic discovery of cancer predisposition genes using the two-hit hypothesis. **a** Knudson’s two-hit model. **b** Principal components analysis (PCA) and clustering using common variants to stratify the population of cancer patients. **c** ALFRED test 1 quantifies the enrichment of rare damaging germline variants (RDGVs) in samples with putative LOH events (estimated via allelic imbalance, AI) using randomization within the PCA clusters (*N* = 10) to control for population structure. **d** ALFRED test 2 quantifies the enrichment of putative LOH events where the RDGV frequency increases (≥10% excess in tumors over normal samples) in samples with AI, using a binomial test. The ALFRED *P*-value is the less significant *P*-value of the two tests. **c** and **d** show the example data for *BRCA1* in a pan-cancer analysis

To systematically identify CPGs from cancer genome data, we devised a statistical method termed ALFRED (for allelic loss featuring rare damaging) that tests Knudson’s two-hit hypothesis genome-wide (Fig. 1 and Supplementary Fig. 1a).

To predict loss of heterozygosity (LOH) in each tumor from exome sequencing data, ALFRED uses all germline variants in coding and noncoding regions within each gene with sufficient sequencing coverage (expanding the analyzed region to 100 kb for genes shorter than this size) and then tests for allelic imbalance (AI), a change in variant allele frequencies (VAFs) in the tumor compared to in the matched non-tumor sample from each patient, Supplementary Fig. 2, see Methods).

ALFRED classifies germline variants (identified from non-tumor DNA; mainly from blood) as potentially damaging if they have a minor allele frequency (MAF) <0.1% in the Exome Aggregation Consortium (ExAC) database^14^ and result in a premature stop codon, frameshift, splice site inactivation, or missense change predicted as deleterious by the MetaLR consensus algorithm^15^.

ALFRED performs two tests using these rare damaging germline variants (RDGVs) and using putative LOH events, which were inferred via AI between the tumor sample and a matched normal sample (see Methods). The first test is for an excess of RDGVs in a gene in tumor samples with putative LOH of the gene, compared to the frequency of RDGVs in the samples without LOH in that gene. This test uses a stratified randomization procedure to account for population structure. The second test is for the direction and magnitude of AI, testing for an increase in the frequency of the RDGV allele in the samples with AI (see Methods). We conservatively use the less significant *P*-value of these two tests as the final ALFRED *P*-value (Fig. 1).

### Application of ALFRED to ~10,000 human tumors

We applied ALFRED to 10,043 tumor exomes from 30 cancer types sequenced as part of The Cancer Genome Atlas (TCGA) project (Supplementary Data 1). The frequency of AI varied widely across samples and tumor types with a median of 7.1% of genes affected in each tumor by our estimates (Supplementary Figs. 4 and 5a and Supplementary Data 2). Ovarian carcinoma (OV) had the highest frequency of AI (median = 17.8% of genes affected, first quartile (Q1) = 15.2%, third quartile (Q3) = 21.1%). Lung squamous cell carcinomas (LUSCs) had the second highest frequency (13.7%), while kidney renal clear cell carcinoma (KIRC, 3.2%), prostate adenocarcinoma (PRAD, 2.4%), and thyroid carcinoma (THCA, 1.1%) had the lowest number of genes affected per tumor (Supplementary Fig. 5a).

We first applied ALFRED in a pan-cancer analysis using all 10,043 samples and testing for an enrichment of RDGVs in samples with AI for the 2983 genes carrying at least five RDGVs (of which at least one with ≥10% increased VAF in tumor compared to matched normal sample) and with an above-average (10%) frequency of AI in the gene in the complete data set (see Methods; Supplementary Data 3).

**Fig. 2:**
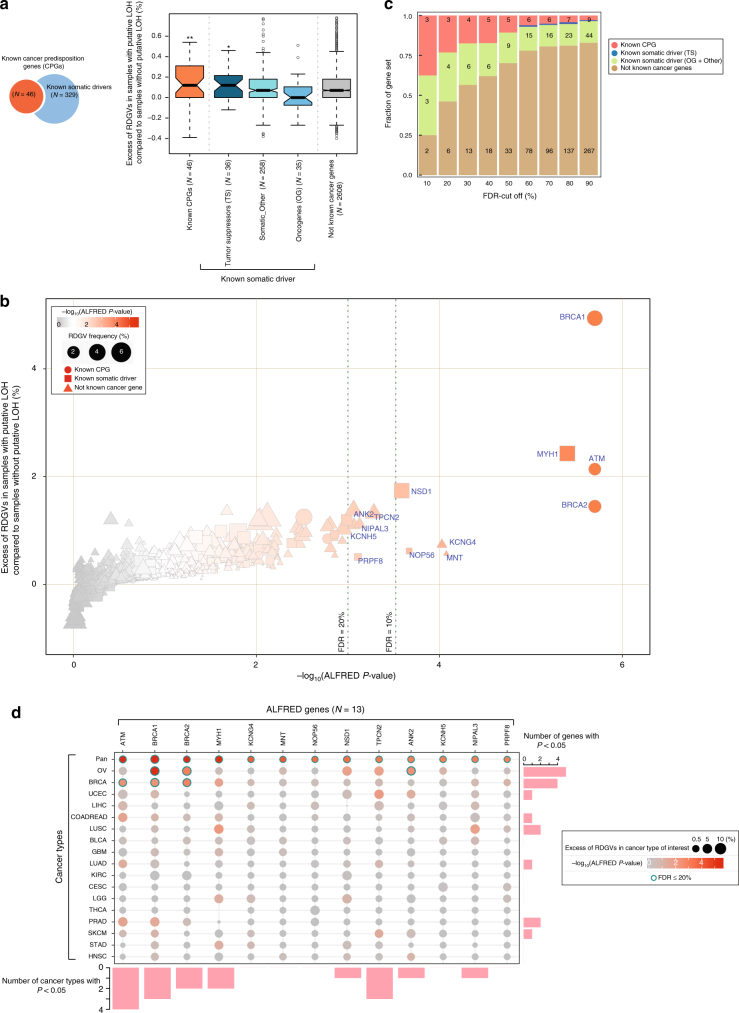
Pan-cancer and 17 individual cancer type ALFRED analyses. **a** Enrichment of RDGVs in samples with AI over samples without AI for different gene sets (*P < 5.0 × 10^−2^, **P < 5.0 × 10^−3^). The median value of each gene set is displayed as a band inside each box. The length of each whisker is 1.5 times the interquartile range (shown as the height of each box). Values lying outside the whiskers are considered outliers. **b** Excess of RDGVs in samples with AI plotted against the ALFRED randomization test significance for individual genes. Color indicates significance and shape represents the type of gene. **c** The fraction of different types of gene in the detected genes at varying FDR cut-offs. **d** ALFRED results in individual cancer types. Enrichments and *P*-values are shown in each cancer type for all genes significant at FDR = 0.2 in the pan-cancer or in the individual cancer type ALFRED tests. Circle size indicates excess of RDGVs within a cancer type and color represents the significance (*P*-value). Blue-border circles indicate genes that are significantly enriched (FDR ≤ 0.2). The number of cancer types in which each gene has *P* < 0.05 and the number of genes with *P* < 0.05 in each cancer type are presented in the bar plots

We first observed that previously known CPGs gathered from a recent literature review^2^ and from the Cancer Gene Census^10^ showed a significant enrichment of RDGVs in samples with AI compared to samples without AI (*P* = 2.2 × 10^−3^ by Mann–Whitney test; Fig. 2a). One example of this is the genes causing Lynch syndrome (a deficiency in DNA mismatch repair), which are robustly enriched as a set (*P* = 3.6 × 10^−3^ by Mann–Whitney test). This extends to DNA repair genes in general (*P* = 3.7 × 10^−2^ by Mann–Whitney test; Supplementary Fig. 7a).

At a false discovery rate (FDR) = 0.2, 13 genes were individually enriched for RDGVs in tumors with AI and exhibited AI in favor of the variant allele (henceforth referred to as ‘ALFRED genes’) (Supplementary Data 3). These 13 genes included three well-known CPGs: *BRCA1* (relative risk (RR) for the excess of AI events in samples with RDGVs compared to without RDGVs = 3.74, 42.2% of patients with RDGVs also have AI versus 16.0% of patients without RDGVs that have AI, ALFRED *P* < 2.0 × 10^−6^), *ATM* (RR = 2.98, 32.8% versus 13.8%, *P* < 2.0 × 10^−6^), and *BRCA2* (RR = 2.25, 37.6% versus 21.5%, 2.0 × 10^−6^) (Fig. 2b; Supplementary Fig. 5c–f). The RDGVs in the 13 ALFRED genes were mainly contributed by deleterious missense mutations (mean of 80%; Supplementary Fig. 8a). The enrichment for known CPGs in this set of 13 genes is very strong (odds ratio (OR) = 29.7, Fisher’s exact test *P* < 3.99 × 10^−4^), demonstrating that, despite LOH potentially being selected for in tumors for multiple reasons^16^, specifically testing for the combination of LOH and RDGVs can identify putative new CPGs without the use of sequencing data from control individuals (Fig. 2c).

We also used ALFRED to analyze each of the 17 cancer types with >300 samples in isolation (82% of the samples in total, Supplementary Data 4). Four genes (six associations: *BRCA1*, *BRCA2*, and *ANK2* in ovarian cancer, *BRCA1*, *BRCA2*, and *ATM* in breast cancer) were significant in at least one individual cancer type (FDR = 0.2, referred to as ‘individual cancer ALFRED genes’) and all four genes were also significant in the pan-cancer analysis (Fig. 2d and Supplementary Data 4). *BRCA1* and *BRCA2* were significant genes in ovarian cancer (*BRCA1*, RR of AI events in samples with RDGVs compared to without RDGVs = 23.3, 94.1% of patients with RDGVs also have AI versus 53.1% of patients without RDGVs, *P* < 2.0 × 10^−6^; for *BRCA2*, RR = 4.7, 83.3% versus 49.3%, *P* < 9.3 × 10^−4^) and in breast cancer (*BRCA1*, RR = 2.5, 48.6% versus 26.6%, *P* < 4.6 × 10^−3^*; BRCA2*, RR = 3.6, 57.1% versus 27.9%, *P* < 1.3 × 10^−3^). Another known cancer susceptibility gene, *ATM*, was also detected in breast cancer^17^ (RR = 2.96, 52.2% versus 26.3%, *P* = 3.5 × 10^−3^).

We observed similar results when examining only rare protein truncation variants (PTVs, encompassing splicing variants, frameshift indels, and nonsense variants) (Supplementary Fig. 8). Five genes were enriched for rare PTVs in tumors with AI and exhibited AI in favor of the variant alleles, of which three genes (*BRCA1/2* and *ATM*) overlap with our initial ALFRED design (RDGVs based model), while *TNFSF13B* (excess of rare PTVs in AI samples over samples without AI samples = 0.37%, PTV-ALFRED *P* = 1.85 × 10^−3^) and *ACACB* (excess = 0.41%, PTV-ALFRED *P* = 3.02 × 10^−3^) are newly detected (Supplementary Fig. 8b). Previously known CPGs also showed a significant enrichment of rare PTVs in samples with AI compared to samples without AI (*P* = 3.7 × 10^−2^ by Mann–Whitney test, Supplementary Fig. 8c). Furthermore, we also used a PTV-ALFRED model to analyze each of the 17 cancer types and three genes were significant in at least one individual cancer type (FDR = 0.2, four associations: *BRCA2* and *ATM* in breast cancer, *BRCA1* and *BRCA2* in ovarian cancer) (Supplementary Fig. 8e and g).

Sub-sampling and repeating the pan-cancer analysis revealed that the number of significant genes increases with the number of samples (*R*^2^ between square root of sample size and number of ALFRED genes = 0.8, Supplementary Fig. 9e), suggesting that many more ALFRED genes will be discovered as more cancer samples are analyzed.

### Somatic cancer genes also carry germline risk variants

We next tested whether cancer genes identified by recurrent somatic alterations but not previously reported to harbor inherited risk variants also showed evidence of carrying recessive RDGVs that predispose to cancer via a two-hit mechanism. Somatic cancer genes known to act via gain-of-function alterations (oncogenes; OGs) showed no significant enrichment for RDGVs in samples with AI (Fig. 2a). In contrast, and consistent with the two-hit hypothesis, somatic cancer genes classified as TSs showed an enrichment for RDGVs in samples with AI (*P* = 2.2 × 10^−2^ by Mann–Whitney test, Fig. 2a). This enrichment was robust when analyzing somatic drivers reported by different studies and more strongly enriched in higher-confidence TSs that were reported in multiple data sets (*P* = 3.08 × 10^−3^ by Mann–Whitney test; Supplementary Fig. 7a, b). This indicates that multiple genes currently only known to be affected by somatic alterations also contribute to cancer because of rare, damaging germline variants.

At an FDR = 0.2, four genes previously reported as somatic cancer genes were significantly enriched for AI in samples with RDGVs in the pan-cancer analysis (OR = 5.1, Fisher’s exact test *P* = 2.1 × 10^−2^; Fig. 2c): *MYH1* (RR = 2.3, 25.3% versus 12.6%, *P* < 2.0 × 10^−6^), *NOP56* (RR = 4.6, 50% versus 17.8%, *P* < 2.12 × 10^−4^), *NSD1* (RR = 2.0, 22.2% versus 12.4%, *P* < 2.56 × 10^−4^), and *PRPF8* (RR = 3.2, 57.1% versus 29.1%, *P* < 7.76 × 10^−4^).

### Germline variants in ALFRED genes increase cancer risk

**Fig. 3:**
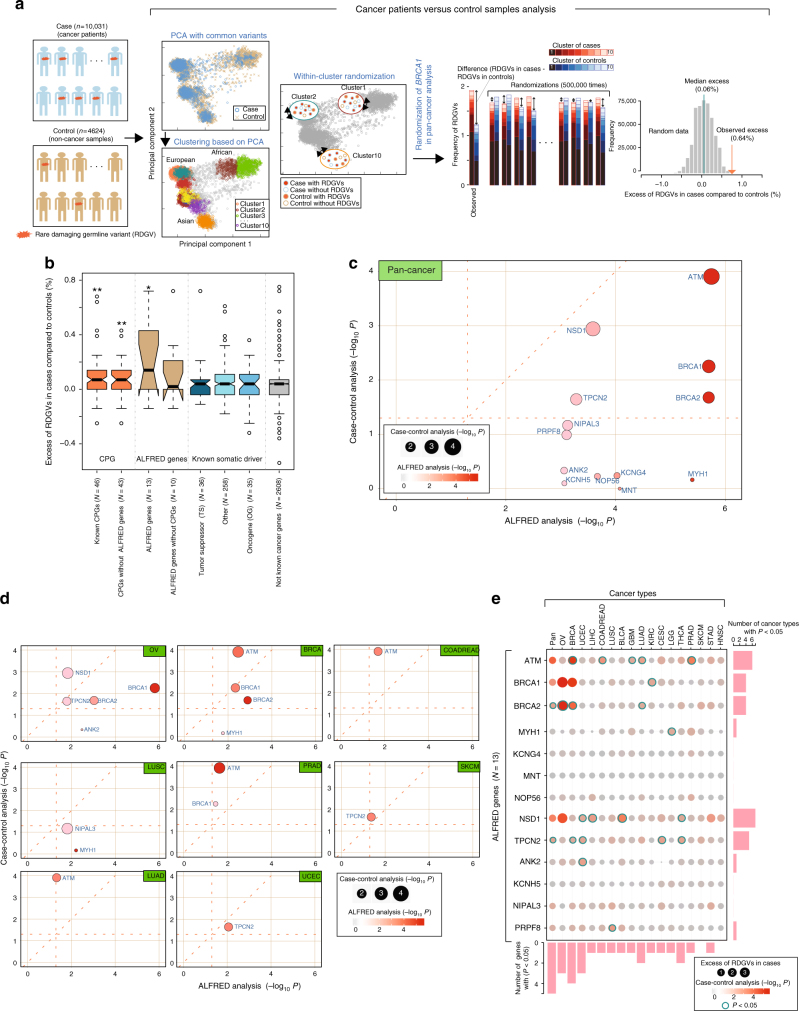
Case–control analysis. **a** Overview of the case–control analysis and randomization procedure used to control for population structure. **b** Enrichment of RDGVs in 10,031 cancer cases over 4624 controls for eight gene sets (*P < 5.0 × 10^−2^, **P < 5.0 × 10^−3^). The median value of each gene set is displayed as a band inside each box. The length of each whisker is 1.5 times the interquartile range (shown as the height of each box). Values lying outside the whiskers are considered outliers. **c** Pan-cancer case–control *P-*values for ALFRED genes. **d** Case–control analyses for eight individual cancer types. **e** Enrichment of RDGVs in cancer patients compared with control samples. Bubble plot shows significance by case–control analysis within each cancer type as a –log_10_ *P*. Circle size indicates excess of RDGVs within a cancer type and color represents the *P*-value. Blue-border circles indicate genes that are significantly enriched (*P* < 0.05). The number of detected cancer types (at *P* < 0.05) in each gene and the number of detected genes (at *P* < 0.05) in each cancer type are presented in the bar plot

We next compared the frequencies of RDGVs in the 13 ALFRED genes in 10,031 cancer patients to the frequencies in 4,624 control exomes compiled from three different studies (see Methods; Supplementary Fig. 1b)^18–20^. We again used a randomization procedure to control for population structure, estimated from common variants (Fig. 3a and Supplementary Fig. 3c–e), and, together with additional quality control steps (see Methods), we only considered variants in regions with sufficient sequencing coverage in both cases and controls (Supplementary Fig. 3f).

In a pan-cancer analysis, RDGVs were enriched 8.5-fold in cases compared to controls in the ALFRED genes relative to other genes (4.4-fold after excluding known CPGs from ALFRED genes, Fig. 3b, average excess of RDGV-bearing individuals in cases over controls = 0.23% of the population per each ALFRED gene, *P* < 1.87 × 10^−2^ by Mann–Whitney test, Fig. 3b; Supplementary Table 1). This was similar to the enrichment for RDGVs across all previously known CPGs (average excess per gene = 0.11% of the population, *P* < 2.04 × 10^−4^ by Mann–Whitney test) and similarly so when excluding the three known CPGs that overlapped with ALFRED genes (average excess per gene = 0.12%).

Five of the pan-cancer ALFRED genes (*BRCA1*, *ATM*, *BRCA2*, *NSD1*, and *TPCN2*) were individually significantly enriched for RDGVs in cases versus controls (*P* < 0.05 by pan-cancer case-control analysis, Fig. 3c) with one additional gene, *NIPAL3*, marginally significant (*P* = 0.07 by case-control analysis) (Fig. 3c). In addition, three of the six individual cancer type ALFRED genes were enriched for RDGVs in cases of the matched cancer type versus controls (*P* < 0.05, Fig. 3d; Supplementary Data 6): *BRCA1* and *BRCA2* in breast invasive carcinoma (BRCA) and OV, and *ATM* in BRCA. Eight of the 13 ALFRED genes with a nominally significant association between RDGVs and AI in at least one cancer type in the ALFRED analysis (*P* *<* 0.05; Fig. 2d and Supplementary Data 5) also had an enrichment of RDGVs in a matched cancer type compared to in controls (*P* *<* 0.05, Fig. 3d; Supplementary Data 6): *ATM* in colon and rectum adenocarcinoma (COADREAD), lung adenocarcinoma (LUAD) and in PRAD, *NSD1* in OV, and *TPCN2* in uterine corpus endometrial carcinoma (UCEC).

We also validated the rare PTV-ALFRED model by comparing the frequencies of rare PTVs in the five PTV-ALFRED genes in cancer patients to the frequencies in control samples. Three of the pan-cancer PTV-ALFRED genes (*BRCA1*, *BRCA2*, and *ATM*) and all four individual cancer type PTV-ALFRED genes were individually significantly enriched for rare PTVs in cases versus controls (nominal *P* < 0.05) (Supplementary Fig. 8f and g).

To evaluate the robustness of this result, we randomly split the TCGA samples into two groups, using one half of the data for the ALFRED analysis (discovery set) and the other half for the case-control analysis (validation set), repeating the split five times. Overall, ALFRED genes presented similar effect sizes to the original ones found on the entire TCGA (Pearson correlation between excess of RDGVs in AI samples ranged from 0.79 to 0.82; Supplementary Fig. 9a) and *P*-values (Pearson correlation between ALFRED −log_10_ *P*-values, 0.71–0.76; Supplementary Fig. 9b). The effect sizes (Supplementary Fig. 9c) and −log_10_ *P-*values (Supplementary Fig. 9d) in the case-control analyses were also highly correlated to the original ones (*R* = 0.88–0.89 and *R* = 0.87–0.89), suggesting robust results.

### Variants in ALFRED genes predispose to specific cancer types

**Fig. 4:**
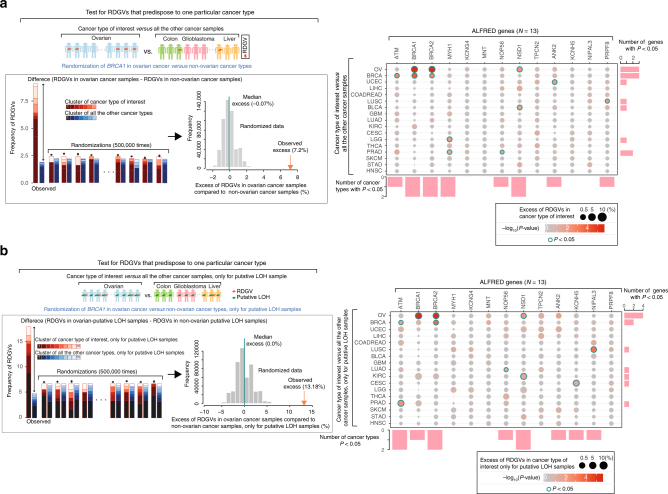
ALFRED genes predispose to specific cancer types. **a** Enrichment and significance of RDGVs in ALFRED genes in each cancer type compared to in all other cancer samples. **b** The same analysis but only considering samples with allelic imbalance at the ALFRED gene locus. Genes and cancer types are ordered as in Fig. 3. Circle size indicates excess of RDGVs within a cancer type and color represents the significance (*P*-value). Blue-border circles indicate genes that are significantly enriched (*P* < 0.05). The number of detected cancer types (*P* < 0.05) in each gene and the number of detected genes (*P* < 0.05) in each cancer type are presented in the bar plot

To further investigate the cancer type-specificity of the cancer risk conferred by rare damaging germline variation in the ALFRED genes, we tested whether RDGVs in these genes were enriched in tumors of one type compared to in all of the other tumor samples (e.g. in ovarian cancer compared to non-ovarian cancer; Fig. 4a). If RDGVs in a gene contribute similar risk to many cancer types then they would not show enrichment in this test. However, if the RDGVs strongly predispose to one or a few cancer types, they should be enriched in patients with these cancer types compared to in other cancer patients. We performed two analyses: the first using all samples and the second restricted to tumor samples with AI in the gene of interest. In total, 8 of the 13 ALFRED genes had an association (unadjusted *P* *<* 0.05) between RDGVs and AI in at least one of the 17 individual cancer types (median 2 cancer types per gene). Four of these eight genes were also significantly enriched overall for RDGVs in the matched cancer type compared to in other cancer types (*P* < 0.05, Fig. 4a; Supplementary Data 7) with six genes enriched when only considering samples with AI (Fig. 4b and Supplementary Data 8). For example, RDGVs in *BRCA1* and *BRCA2* were, as expected, significantly enriched in OV and BRCA compared to in all the other cancer samples (*BRCA1*, excess of RDGVs in BRCA compared to non-breast cancer = 2.1%, 95% CI: 1.1–3.1%, excess in OV = 6.7%, 5.1–8.2%; *BRCA2*, excess in BRCA = 1.3%, 0.49–2.1%; excess in OV = 3.9%, 2.7–5.1%).

In total, therefore, seven of the eight ALFRED genes with a nominally significant association between RDGVs and AI in at least one cancer type in the ALFRED analysis also had a significant enrichment (unadjusted *P* < 0.05) of RDGVs in that cancer type over either control samples or other cancer types (Supplementary Data 9**)**. Moreover, four genes had a significant enrichment in both additional RDGV frequency analyses (*BRCA1*, *ATM*, *BRCA2*, and *NSD1*).

### The contribution of ALFRED genes to cancer risk

**Fig. 5:**
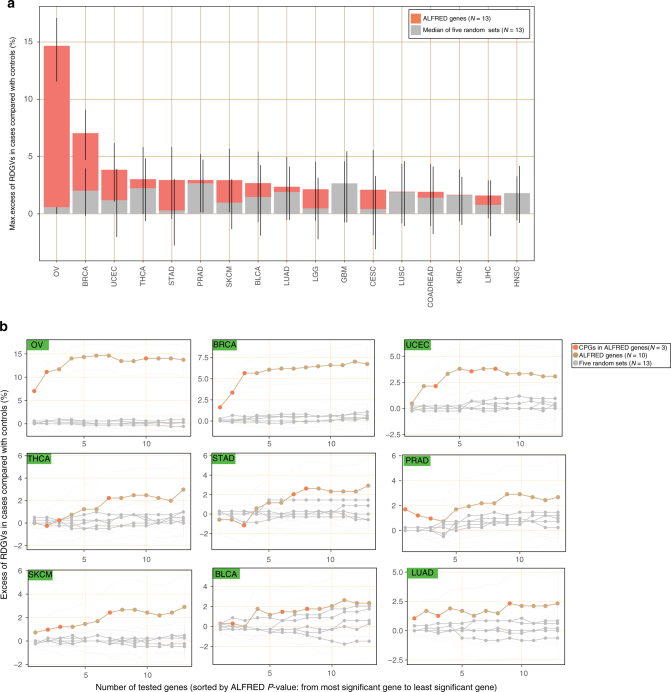
Contribution of ALFRED genes to cancer risk. **a** Maximum excess of RDGVs in cases compared to controls adding genes sequentially according to their ALFRED *P*-value. Genes are randomly ordered in the random sets. Excess was calculated using a randomization for ALFRED genes (colored) and five random gene sets of the same size (gray). Error bars indicate 95% confidence interval. **b** Results for the nine cancer types with largest maximum enrichment

Next, we estimated the total contribution of RDGVs in the ALFRED genes to cancer risk by quantifying the excess frequency of ALFRED gene RDGVs in cancer patients over that in the general population. Again, this was adjusted for the expectation based on the population structure, as determined by a randomization test (Methods). We examined ALFRED gene sets at different stringency thresholds, and quantified the excess frequency of RDGV-bearing cases (cancer patients) while adding genes sequentially according to their ALFRED *P*-values for each cancer type (ordered from the most significant gene to least significant gene; Fig. 5), reporting the maximum excess of individuals carrying RDGVs in cases compared to controls. This was significantly greater than the random expectation in 5 out of 17 individual cancer types (Fig. 5 and Supplementary Fig. 10a).

The estimates of contribution to cancer risk were markedly different across cancer types with a median excess of individuals with RDGVs in cases compared with controls = 2.3% and a range of 1.4% (head and neck squamous cell carcinoma (HNSC)) to 14.6% (OV). Strikingly, 21.7% of OV patients carried RDGVs in ALFRED genes, which is an excess of 14.6% over controls (95% CI: 11.6-17.1%). Other cancer types with a substantial contribution of RDGVs in ALFRED genes include BRCA (7.0% by excess of cases versus controls, adjusted to random expectation; 95% CI: 4.7–9.1%) and UCEC (3.8% excess, 95% CI: 1.1–6.2%).

We next compared the cancer risk contribution of RDGVs in the ALFRED genes to the contribution of RDGVs in previously reported CPGs (Supplementary Fig. 10). We first focused on the contribution of RDGVs in the three previously known CPGs that were also retrieved by ALFRED (*BRCA1*, *BRCA2*, and *ATM*). The excess of RDGVs in these three CPGs in cases versus controls suggests that RDGVs in these three genes are implicated in a median of 1.2% of cancer cases across the 17 cancer types (range: 0.24–11.4%). However, RDGVs in the remaining ten newly discovered ALFRED genes were estimated to explain a median of 1.8% of cases across cancer types (range: 0.32–4.0%). In OV, for example, the excess of cancer cases that carry RDGVs in any ALFRED gene after excluding known CPGs is 4.0% (95% CI: 1.6–5.0%). Similarly, for four other cancer types (bladder urothelial carcinoma (BLCA), PRAD, THCA, and UCEC), the ten putative novel ALFRED genes are estimated to explain approximately 2% of cancer cases. For comparison, the percentage of cancer cases explained by a general set of 46 previously reported CPGs is 4.9% (median across cancer types; range 0.8–11.7%). However, the CPGs known to predispose specifically to individual cancer types were estimated to contribute to 1.0% of cases (median across cancer types; range 0–11.4%, Supplementary Fig. 10b). The newly discovered ALFRED genes therefore appear to contribute more cancer risk than the previously known CPGs relevant for each cancer type.

To estimate the total proportion of cancer cases attributable to rare germline risk variants for each cancer type, we combined the ALFRED genes with the previously reported CPGs (for any cancer type). In total, RDGVs in these 56 genes explain a median of 5.4% of cancer cases across the 17 cancer types (excess frequency of cases with a RDGV over frequency of controls, adjusted to a random expectation; range 2.3–15.2%). For instance, a total of 15.2% (95% CI: 12.1–17.7%) of OV and 9.3% of BRCA cases (95% CI: 6.2–12.4%) can be explained by RDGVs in the 56 genes (Supplementary Fig. 10b).

## Discussion

The two-hit hypothesis has served as a framework for cancer gene discovery for over 40 years^3,4^. Here we have shown that this classic insight still provides a powerful hypothesis for the discovery of CPGs and, in particular, that it can be used to discover CPGs from cancer exomes without the use of control samples.

Only three of the genes identified by ALFRED (*BRCA1*, *BRCA2*, and *ATM*) are known CPGs reported in two large-scale literature surveys of CPGs identified through family studies^2,10^. Our results suggest that multiple somatic cancer drivers and putative new genes also harbor germline genetic variants that predispose to cancer in the general population. For example, the histone H3 lysine 36 methyltransferase *NSD1* was the second most significantly enriched gene in our case–control analysis with an excess of RDGVs in cases compared with controls = 0.72% (*P* < 1.14 × 10^−3^, 95% CI: 0.27–1.2%). This suggests that RDGVs in *NSD1* are causally implicated in ~0.72% of cancers, a similar magnitude of effect as we observe for the well-known cancer predisposition genes *BRCA1* (0.64%) and *ATM* (0.68%). Genome sequencing has previously established *NSD1* as a somatically mutated cancer driver in HNSC^21^ and LUSC^22^, and recurrently silenced by methylation in renal cell carcinoma^23,24^. Here we have presented evidence that *NSD1* also carries germline cancer predisposition variants. Loss-of-function germline variants in *NSD1* cause Sotos syndrome, a rare genetic disorder characterized by tissue overgrowth during the first years of life^25^. However, the variants in *NSD1* enriched in cancer patients are distinct from the variants that cause Sotos syndrome (Supplementary Fig. 12h) and they are much less likely to be truncation variants (OR = 151.8, Fisher’s exact test *P* < 2.1 × 10^−40^), suggesting different mechanisms or allele-strengths underlie cancer predisposition and Sotos syndrome.

Considered as a set, RDGVs in the ALFRED genes can explain a substantial proportion of the cancer cases analyzed by the TCGA project: a median of 2.3% across the 17 individual cancer types with sufficient sample sizes. However, in several cancers the contribution is substantially higher, with 14.6% of OV, 7.0% of BRCA, and 3.8% of UCEC cases attributable to RDGVs in these genes. Including additional known CPGs further increases the estimate of the proportion of cases attributable to RDGVs: a median of 5.4% across the 17 individual cancer types, with 15.2% of OV, 9.3% of BRCA, and 6.0% of UCEC cases attributable to RDGVs in ALFRED genes, respectively.

The sequencing of even larger numbers of tumors and control individuals will further refine these estimates (Supplementary Fig. 9e) and will also allow a more complete description of the genes that contribute to cancer when they are inactivated by the combination of RDGVs and somatic second hits.

## Methods

### Ethical approval

This paper reanalyzes previously published data sets. All cancer patient and healthy controls data were handled in accordance with the policies and procedures of the Centre for Genomic Regulation (CRG).

### Tumor exome sequences

The whole-exome sequences of TCGA cancer patients were downloaded from the Cancer Genomics Hub repository (https://cghub.ucsc.edu/)^26^. A pair of BAM files per person was obtained: one with aligned short reads derived from the healthy tissue (commonly, blood) of the donor, and another from the tumor sample from the same person. The corresponding BAMs are available from TCGA following authorization (dbGaP controlled data set phs000178). Most of these BAMs (*N* = 9,774) were pre-aligned to the hg19 assembly. For the remaining 637 samples aligned to hg18, we realigned the reads to hg19 using Illumina’s Isaac Aligner v1.14 (ref. ^27^) with default parameters, except for specifying “--use-bases-mask Y75,Y75” if the aligner run would not complete at default settings. Clinical data were downloaded directly from the TCGA Data Portal (https://portal.gdc.cancer.gov). Technical covariates of TCGA samples (*N* = 9618) were obtained from Buckley et al.^28^.

### Control exomes

The exome sequences of healthy controls were collected from the 1000 Genomes Project^19^ (1000g; Phase III high-coverage whole-exome sequences, the European (*N* = 500), East Asian (*N* = 513), African (*N* = 596), and Admixed American (*N* = 345) populations; total *N* = 1954 exomes), from the Women’s Health Initiative (WHI; *N* = 791 European American, *N* = 614 African American, and *N* = 3 of unknown ethnicity; in total, 1408 samples (dgGaP phs000200) (https://esp.gs.washington.edu/drupal/)^20^ and from the UK10K data for *N* = 1658 samples (http://www.uk10k.org/)^18^.

### Sample-level quality control and genomic region filtering

To ensure sufficient sequencing coverage, we required that all genomic sites retained for further analysis have ≥8 reads covering a site in at least 90% of the cancer samples in each cohort (90 out of 100 randomly chosen samples). The threshold of 8 reads was imposed after having applied the built-in read quality filters of Illumina’s Isaac Variant Caller (IVC) software v1.0.6, which was run using default settings^27^. Within the TCGA set of cancer cases we considered sequencing centers (BI, WU, and BCM) separately for the purposes of this analysis, meaning that there needs to be sufficient sequencing coverage in ≥90% of the samples from each of the three centers for that genomic site to be allowed. Moreover, we similarly subdivided the control data sets, requiring ≥8 high-quality reads in at least 90% of samples from each of the 1000 Genomes sequencing centers (BI, WU, BCM, and BGI) independently. The WHI was considered as a single unit in this analysis but was filtered to exclude exome sequences with low overall sequencing coverage, thereby retaining 1023 WHI samples and examining only the sites with ≥8 high-quality reads in 90% of a randomly sampled set of those tumors (Supplementary Fig. 3f). Finally, from the UK10K cohort, we randomly selected three studies (EGAD431, EGAD433, and EGAD438) and required sufficient sequencing coverage in 90 of 100 exomes from each of these. Thus, in total, we made 11 genome masks (3 for TCGA sequencing centers, 4 for 1000g, 1 for WHI, and 3 for UK10K) and intersected them to arrive at the final set of allowed genome regions for the case–control analysis, which spans 33.82 Mb of the hg19 reference. This encompasses 14,143 genes that therefore have sufficient sequencing coverage in both TCGA and the control samples. After filtering to retain only the validated variants in ExAC version 0.3 (ref. ^14^) (http://exac.broadinstitute.org/), the TCGA cancer samples had a median 4574 nonsynonymous, stop gain and stop loss, splice SNVs and coding indels in the filtered regions, as annotated by the Annovar tool version 2014-11-12 (ref. ^29^), while the control samples had a median 4588 variants each, according to the same definition.

Importantly, the LOH calling procedure (estimated from AI between the tumor and the normal sample from cancer patients; see below) were performed only on the TCGA samples and not on controls, allowing us to use a less stringent definition of covered genomic regions specifically for the purposes of determining LOH. This was obtained as an intersection of only the three TCGA genomic masks, thus covering 50.37 Mb of genomic DNA and affording more coverage at the noncoding intronic and intergenic sites that flank exons. The TCGA cancer samples had a median of 5154 variants (21,780 all germline variants both coding and noncoding variants) in the covered regions.

### Sample filtering

Before proceeding with further analyses, we removed (1) a set of 222 TCGA samples sequenced with the ABI platform that were outliers in a principal components analysis (PCA) analysis and (2) the bottom 2% of samples with the lowest number of called nonsynonymous variants (*N* = 146 TCGA samples in the ALFRED analysis; *N* = 169 including 11 control samples in the case–control analysis).

### Calling germline variants

We called the germline variants (single-nucleotide and short indels) on the normal and the tumor samples independently using Illumina IVC^27^. We used the default IVC confidence threshold (genotype quality score GQX ≥ 30) on the normal samples to determine the germline variants. Furthermore, we discarded all indel variants covered with less than 10 reads and when the allelic frequency was significantly different from 50% and also from 100% in the normal sample (Chi-square test *P* > 0.05).

### Variant annotation and filtering

We annotated the called variants in the VCF files using Annovar^29^, database version 2014-11-12. Of the data Annovar reports, we used (i) the consequences of the mutations: synonymous, missense, truncating, splice site, frameshift indel, and in-frame indel, using the RefSeq gene annotations^30^; (ii) the estimated effect of missense mutations via the MetaLR predictor^15^, which combines nine deleteriousness scores including PolyPhen-2, SIFT and others. We discarded all variants marked as possible artifacts in the ExAC (via VQSR recalibration scores supplied therein) or that were completely absent from ExAC. This filtering was performed on the full ExAC, which includes germline variants of TCGA samples in addition to other non-cancer cohorts. We also discarded double-nucleotide variants annotated by ExAC. Finally, we compared detection frequencies of common variants (MAF > 5%) across TCGA and three different control data sets. All pairwise combinations show very strong correlations (Pearson correlation ranges from 0.92 to 0.99), suggesting that no major sequencing artifacts were observed in our analysis (Supplementary Fig. 3c).

### Detecting putative LOH events

In order to determine whether LOH has occurred in each gene in each tumor sample, we considered all germline variants (both rare and common), taking into consideration both the coding and noncoding (intronic/UTR) variants. The average number of variants of gene per sample is highly correlated with gene length (Pearson correlation coefficient (PCC) = 0.55; Supplementary Fig. 2a). To reduce biases this may introduce, we added neighboring variants: (1) within 100 kb and (2) extending the window to 200 kb. The length bias is much reduced after adding neighboring variants within 100 kb ( PCC = 0.25). Employing an even longer window size (200 kb) does not further appreciably reduce the correlation between gene length and number of variants (PCC = 0.18). In conclusion, we reasoned that adding neighboring variants is warranted in order to lessen the bias wherein longer genes provide more statistical power to detect LOH and that a window size of 100 kb is sufficient since increasing the window size further is not advantageous.

When testing genes shorter than 100 kb, we extended the examined region bidirectionally so as to ensure that the gene was represented by variants spanning at least 100 kb across the chromosome. In the case when a gene is longer than 100 kb, we only considered the variants within that gene but without extending to include the neighbors. Similarly to calling coding variants, we also limited the analyses to genomic regions with sufficient sequencing coverage in the TCGA samples (see above). Homozygous germline mutations were not included in further analyses. Before performing a statistical test to call LOH, we applied an effect size threshold, requiring that the tumor VAF of a germline variant must be either higher than 0.7 or lower than 0.3. This ensures that the LOH was not a late event during tumorigenesis, which is an unlikely scenario for an LOH event associated with cancer-predisposing germline variants. Each variant in a gene (and possibly surrounding regions) that meets the effect size threshold was further tested individually using a two-tailed Fisher’s test that compares the read counts supporting the variant and the reference alleles in the tumor, versus the read counts supporting the variant and the reference alleles in the normal (noncancerous) tissue. The *P*-values from all tested variants corresponding to the gene were then pooled using Fisher’s method for combining *P*-values. Finally, we called LOH in the gene if the nominal pooled *P*-value was ≤0.05. Applying this cutoff provides putative LOH labels that are further used as input for the ALFRED test (see below) that, in turn, provides FDR-adjusted statistical significance estimates.

We compared our AI detection method to copy number changes reported using an independent method (GISTIC analysis of Affymetrix 6.0 SNP array data by Broad Firehose analysis pipeline^31^ (http://gdac.broadinstitute.org/)) applied to 9672 TCGA samples. We compared our classification (AI or non-AI) to their copy number alteration (CNA) categories—(i) loss, (ii) neutral, or (iii) gain—for all tested genes. Our AI events were classified as losses (44.9%), neutral (31.7%), and gains (23.4%), which compares to 15%, 67.7% and 23.4%, respectively, for non-AI events.

### Rare damaging germline variants

Rare variants were defined as those whose frequency was <0.1% in each of the six subpopulations: African/African American (AFR), Latino (AMR), EAS (East Asian), Finnish (FIN), Non-Finnish European (NFE), and South Asian (SAS) and also globally in ExAC. Damaging variants were defined as splicing variants, frameshift indels, nonsense variants, and deleterious missense variants annotated as “D” (deleterious) by the MetaLR predictor^15^. Additionally, we removed the RDGVs that were recurrent at the same position in more than 1% of our samples (TCGA or control samples), thereby excluding four variants (17-46608203-A-G, 20-5548206-TC-T, 21-34924148-A-G and X-2833605-C-T).

### Pan-cancer ALFRED analysis

We first tested for an excess of RDGVs in samples with putative LOH compared to in samples without putative LOH of all possible genes (*N* = 14,143), collapsing together all SNVs/indels in each gene in each sample and using the exomes of all 30 cancer types (Supplementary Fig. 5b; Supplementary Data 1). To increase statistical power, we first restricted our analysis to the genes with high frequency of putative LOH events (above average in our data set, 10.0%). Next, we applied a threshold fornumber of RDGVs that ensures there is no inflation in the distribution of observed *P*-values (see below), implying a statistically well-calibrated test. Finally, 2983 genes were defined as ALFRED tested genes, which carried at least five RDGVs (of which at least one with ≥10% increased VAF of RDGVs in tumor compared to matched normal sample; 6692 genes were excluded that were carrying less than five RDGVs and, additionally, 329 genes were excluded if they carried no RDGV with ≥10% increased VAF), with an above-average (10.0%) frequency of putative LOH in the gene in the pan-cancer data (8809 genes were excluded that were lower than 10.0% frequency of putative LOH; 4672 genes were carrying less than five RDGVs and lower putative LOH frequencies than average frequency of putative LOH; Supplementary Fig, 6a). With these criteria, the tested ALFRED genes were biased towards larger genes because larger genes tend to present higher AI and RDGV frequencies (PCC = 0.40 between length and RDGV frequency, PCC = 0.23 between length and LOH frequency; Supplementary Fig. 6b). However, the ALFRED method is designed to test for the co-occurrence of LOH events and RDGV, and we observed only a very weak correlation between ALFRED *P*-value (as −log_10_ *P*) and gene length (PCC = 0.09).

To determine the significance of the excess of RDGVs in putative LOH samples compared to in samples without putative LOH, we applied a randomization procedure that controls for the population stratification by randomizing the labels within subpopulation clusters determined by PCA analysis (see below, Controlling for population structure in the randomization test).

To evaluate the influence of known CPGs on the identification of ALFRED genes, we performed an FDR correction only considering a set of known somatic cancer genes (meaning, the known germline CPGs were excluded). We detected five genes at FDR 20%, of which four genes (*MYH1*, *NSD1*, *NOP56*, and *PRPF8*) overlap with our initial design, and additionally *INO80* (ALFRED *P* = 1.03 × 10^−3^) was newly detected. This supports the notion that ALFRED analysis could identify putative novel genes even without including known CPGs.

To ensure the robustness of our ALFRED method, we considered possible confounding factors. Several biological features of the tumors could affect our LOH estimates, such as genomic instability, burden of RDGVs, sample purity, ploidy, intra-tumor heterogeneity, and patient age (see section below, Associating AI frequency with biological features). We evaluated the association between these factors and the frequencies of our AI calls or RDGV frequencies, but found no evidence of confounding (Supplementary Fig. 11). While AI frequencies do show positive correlations with genomic instability (PCC = 0.34), ploidy (PCC = 0.36), and patient age (PCC = 0.11) in the pan-cancer analysis, the ALFRED analysis—which considers the overlap of RDGVs and AI events—does not appear to be overly affected by these biological features (Supplementary Fig. 11c–e).

The ALFRED method was also robust to other possible confounders: (1) sequencing artifacts due to whole-genome amplification (in OV and LAML samples; Supplementary Fig. 12a), (2) somatic second hits not due to LOH but due to somatic truncation mutations (Supplementary Fig. 12b; Supplementary Data 11), (3) apparent LOH events which might be due to amplification of the wild-type allele (Supplementary Fig. 12f), and (4) presence of haploinsufficient genes (Supplementary Fig. 12g). In all such cases, the distributions of –log_10_ *P*-values of the ALFRED test were stable (PCC between original and filtered data sets = 0.83 to 0.97, see Supplementary Fig. 12a, b and f).

We also explored the possibility that the ALFRED analysis could identify genes with dominant gain-of-function variants. Genes with dominant gain-of-function variants would not be significant in ALFRED analysis (e.g., no excess of RDGVs in AI samples compared to no-AI samples), but would be significant in the case–control analysis, meaning the variants are enriched in cancer patients in comparison to the general population. We observed that 44 genes presented low effect size of ALFRED analysis (RR for the excess of putative LOH events in samples with RDGVs compared to without RDGVs, RR < 1.0), but were nominally significant in the case-control analysis (unadjusted *P* < 0.05). There are four somatic drivers (not classified as TSs or OGs) and one OG (*JAK2*, RR = 0.98, case–control *P* = 9.28 × 10^−4^) detected (genes are labeled in Supplementary Fig. 12e). While our initial ALFRED analysis was not explicitly designed to identify cancer predisposition genes with dominant-negative effects, this result suggests that indeed some cancer genes with a dominant effect might be identified in the future by applying custom-developed methods to cancer sequencing data.

### ALFRED analysis of individual cancer types

We performed the same analysis for each of 17 cancer types with >300 samples, a total of 8283 samples (82% of all samples): BLCA, BRCA, cervical squamous cell carcinoma and endocervical adenocarcinoma (CESC), COADREAD, glioblastoma multiforme (GBM), HNSC, KIRC, low-grade glioma (LGG), liver hepatocellular carcinoma (LIHC), LUAD, LUSC, ovarian serous carcinoma (OV), PRAD, skin cutaneous melanoma (SKCM), stomach adenocarcinoma (STAD), THCA, uterine corpus endometrial carcinoma (UCEC). As in the pan-cancer analysis, in each cancer type we required the putative LOH frequency to be above the average putative LOH frequency recorded across all samples (10.0%). To avoid inflation of the *P*-value distribution, we adjusted the requirement for the number of samples carrying a RDGV in each cancer type as follows: more than two case (samples with LOH event) or control samples (samples without LOH event) with a RDGVs in THCA, more than three in five cancer types (CESC, KIRC, OV, PRAD, and UCEC), more than four in GBM, more than five in four cancer types (BLCA, LGG, LIHC, and LUSC), more than six in three cancer types (BRCA, COADREAD, and HNSC), more than seven in SKCM, more than nine in two cancer types (LUAD and STAD).

To check the robustness of ALFRED analysis of single cancer types, we have also confirmed results in the case when FDR correction was performed across the statistical tests in 17 cancer types considered together (in the same manner as for the pan-cancer analysis; Supplementary Fig. 12d and Supplementary Data 10). Four genes (six associations) were detected in at least one individual cancer type after merging all the cancer types together (FDR = 20%) and all four genes overlapped with the genes detected when FDR correction was done in each cancer type separately.

### PTV-only ALFRED analysis

Additionally, we designed a PTV-ALFRED model that tested for an excess of rare PTVs (without considering rare deleterious missense variants) in tumor samples with a putative LOH event over samples without putative LOH in a pan-cancer analysis and in 17 individual cancer types (Supplementary Fig. 8b and e). We restricted our analysis to genes with a high frequency of putative LOH events and defined a threshold of the number of rare PTVs that ensures no inflation and no deflation in the distribution of observed *P*-values (lambda = 0.96). In the pan-cancer analysis, 174 genes were included in the PTV-ALFRED model with at least five rare PTVs (Supplementary Data 12**)**. We also used a PTV-ALFRED model to analyze each of the 17 cancer types and three genes were significant in at least one individual cancer type (Supplementary Fig. 8e; Supplementary Data 13**)**.

### RDGV frequency analyses

To further evaluate the contribution of RDGVs in the ALFRED genes towards cancer risk, we designed three different tests and applied a randomization procedure to each of them in order to determine the statistical significance and the effect size.

1. Cancer patients versus control exomes: we tested for an excess of RDGVs in cases (all TCGA samples) versus controls (the general population). We also performed the same analysis for 17 individual cancer types separately (Supplementary Fig. 6d).
2. Cancer type of interest versus cancer samples of all other cancer types: we tested for an excess of RDGVs in one TCGA cancer type compared to in all the other TCGA samples (e.g., breast cancer versus non-breast cancers) for each of the 17 cancer types (Supplementary Fig. 6e).
3. Cancer type of interest versus all the other cancer types for putative LOH samples only: we tested for an excess of RDGVs in samples of each of the 17 cancer types versus samples of the other cancer types as above but only considering samples with putative LOH at the locus being tested (Supplementary Fig. 6f).

To evaluate the robustness of our second RDGV frequency analysis, we sought to determine whether our analysis could distinguish the cancer type-specific enrichment when some genes predispose to more than one cancer type (e.g., *BRCA1* in OV and BRCA). We have tested this possibility by performing an analysis in which we tested each cancer type of interest versus all the remaining cancer samples, but excluding one of the other types. Then we repeated this analysis for all ‘other’ cancer types one-by-one (e.g., breast cancer versus non-breast cancer types except ovarian cancer; then, breast versus non-breast cancer types except bladder cancer etc.). For each cancer type, we therefore repeated this test 16 times, excluding each one of the remaining types. The distribution of *P*-values with this modified tissue-specificity analysis is rather similar to our initial design (Supplementary Fig. 12c), suggesting the general robustness of our initial analysis. One novel association (*ATM* in COADREAD) becomes nominally significant in this modified tissue-specificity analysis (*P*-value changed from 5.3 × 10^−2^ to 2.7 × 10^−2^). Also, as expected, *BRCA1* in BRCA presented a slightly better supported association in the modified tissue-specificity analysis (breast cancer versus non-breast cancer types except ovarian cancer) (*P* < 2.0 × 10^−6^) compared to the previous analysis (breast cancer versus non-breast cancer types; *P* < 8.4 × 10^−5^).

### Controlling for population structure

Many germline variants from whole-exome or genome sequencing data are expected to vary according to the ethnicities of the individuals within the cohort. This is evident in PCA plots of germline variation^19^ and may confound genome-wide association studies^32^. We thus employed a randomization test that controls for population stratification by comparing matched samples only within subpopulations (Supplementary Fig. 5i; see the Randomization algorithm section), as described in ref. ^11^. Past work using simulated data suggests that such matching controls for *P*-value inflation equally well or better than the approach where the population PCs are included as covariates in regression^13^.

To define the subpopulations in our data, we performed a PCA with only the common germline variants (≥5% MAF in ExAC). For the ALFRED analyses and the cancer type of interest versus all other cancers analyses, we performed the PCA only on the TCGA samples. For the other case–control analyses we performed the PCA on both the TCGA and control samples. We used the first four PCs to cluster the individuals using the tclust package in R (Supplementary Fig. 3a and d)^33^, a robust clustering algorithm that trims outlying samples^34^. We grouped samples into *k* = 10 clusters for both the TCGA-only analysis and also *k* = 10 separately for the TCGA plus controls analysis.

### Stratified randomization algorithm

We aggregated together the RDGVs in each gene^11,12^. Each sample was then assigned as carrying (‘1’) or not carrying (‘0’) at least one of such qualifying variants. To determine the statistical significance of the excess of RDGVs, we applied a randomization procedure to each of the different testing scenarios described above, in which the labels of the individuals are randomized within population strata (clusters determined on principal components of the common variant matrix; see above), but they are not randomized across strata. The labels are (i) in the ALFRED analysis: 1, putative LOH sample, 0, no-LOH sample; (ii) in the case versus control analysis: 1, cancer sample, 0, control sample; (iii) in the cancer type of interest versus all other cancer types analysis: 1, cancer type of interest; 0, all other cancer types; (iv) in the cancer type of interest versus all of the other cancer types analysis only for putative LOH samples: 1, putative LOH samples in the cancer type of interest; 0, putative LOH samples in all other cancer types. In each iteration the test statistic is computed, which is the difference between (i) the relative frequency of samples (individuals) carrying RDGVs in the tumors with putative LOH and (ii) the relative frequency of samples carrying RDGVs in the tumors without putative LOH. Of note, the LOH tumors and the no-LOH tumors can be substituted with cases and controls, respectively, thereby allowing the same randomization procedure to be applied to the case–control analysis; see above for details. In other words, we test for significant excess of the proportion of the RDGV-bearing gene in cancer patients exhibiting putative LOH, or, equivalently, the excess of the proportion of putative LOH-exhibiting gene in samples bearing a RDGV.

We randomized 500,000 times to determine an empirical *P*-value, which is the number of randomizations reporting an equal or higher value of the test statistic for a given gene than was observed in the actual data. We examined the distributions of *P*-values across test genes using quantile–quantile (Q–Q) plots, which indicated no inflation in the individual randomization experiments (lambda ranges from 0.1 to 1.0; Supplementary Fig. 6c). FDRs were calculated using the Benjamini–Hochberg method^35^. In addition to the significance call for each gene, we also report the effect sizes, which are found by subtracting the median test statistic (excess % RDGV-carrying genes) across all randomization iterations from the observed value of the test statistic in the actual data. This effect size quantifies the observed excess of individuals harboring RDGV over a random distribution, while accounting for the population structure. Moreover, we also report the 95% confidence interval (CI) of the effect sizes, whose upper and lower bounds were found by subtracting the 2.5th and the 97.5th percentile of the randomized distribution from the observed value of the test statistic, respectively (Supplementary Data 3).

In addition to testing individual genes, we also tested for significance of a set of ALFRED genes pooled together. These were tested similarly as for individual genes as above, except that here the set of genes in question is effectively treated as a single concatenated gene. In other words, we quantified the relative frequency of individuals harboring a RDGV in any of the genes in the set versus the individuals without RDGVs in any of the genes in the set. The *P*-values, effect sizes, and confidence intervals were calculated as above. The reported effect size can again be interpreted as an excess relative frequency of individuals harboring a RDGV in any of the genes in this set, adjusted for a baseline defined by the population stratification.

### Test for direction of AI

As described above, the first step in the ALFRED method is a test for an excess of RDGVs in samples exhibiting AI. The second step is a test for the direction and for the magnitude of AI that ensures that it is the wild-type allele that is commonly lost, and not the RDGVs. In particular, we quantify the VAF difference of the RDGV between the normal tissue and the tumor sample. If the VAF of the RDGV is increased by ≥10% in the tumor compared to the normal sample, that particular tumor sample is considered to have a putative two-hit event; if the VAF of the RDGV is increased by less than 10% or it is decreased, there is no two-hit event in that tumor. Next, for each gene, we test if there is an enrichment of such two-hit events (where the RDGV increases in VAF ≥ 10%) in AI samples compared to in no-AI. This is determined by using a binomial test (one-tailed), where the baseline relative frequency of the putative two-hit events is determined from their counts in the no-AI samples for that same gene.

Of note, the test for direction of AI additionally imposes a threshold for effect size: at least 10% VAF increase is required, and smaller increases do not count towards the final tally of putative LOH events. This is a conservative filter, since it discards the more subtle increases in VAFs. To empirically estimate the effects of the 10% cutoff, we examined the samples containing rare truncating (nonsense or frameshift indel) variants of six genes that were previously associated with inherited ovarian carcinoma^36^(*BRCA1*, *BRCA2*, *MSH6*, *PALB2*, *RAD51*, and *TP53*) in the TCGA ovarian cancer data (*N* = 51 in our data set); these were the putative true positive LOH events. Then, we randomly shuffled 100,000 times the VAFs between the tumors and matched normal samples, thereby obtaining the empirical distributions for the null hypothesis of no-VAF differences between tumor and normal samples (in effect, we simulated the putatively true negative events). The ≥10% threshold for VAF increase is indeed near-optimal on the receiver operating characteristic curve created using 51 rare truncation variants of the six genes, shown in Supplementary Fig. 5g (sensitivity = 0.92; specificity = 0.74; balanced accuracy = 0.83). A higher VAF increase threshold (≥20%) threshold results in an inflated *P*-value distribution (lambda = 1.3), which is not desirable. Finally, to combine the two ALFRED tests in a conservative manner, we retain the less significant *P*-value of the two tests: (i) the AI-RDGV co-occurrence test and (ii) the AI direction test, thereby obtaining the final ALFRED *P*-value (Fig. 1).

### Estimating the contribution of ALFRED genes to cancer

We prepared seven gene sets to compare the maximum excess of RDGVs in cases compared to controls: known CPGs that were also discovered by ALFRED (*N* = 3), ALFRED genes as a full set (*N* = 13), and without known CPGs (*N* = 10), CPGs known to predispose to particular cancer types from literature (*N* = 1 (CESC) to 11 (GBM))^2,10^, all known CPGs considered as a full set (*N* = 46), and the union of ALFRED genes and the CPGs (*N* = 56), and additionally sets of random genes (Supplementary Fig. 10). For the random control, the same number of genes as for the ALFRED genes (Fig. 5) or the combination of ALFRED genes and CPGs (Supplementary Fig. 10) were randomly selected five times from a general set of genes (*N* = 2983 which were analyzed in the pan-cancer ALFRED analysis; this excludes the ALFRED genes and known CPGs) and calculated the median values of these excesses of RDGVs in cases versus controls. For the three gene sets that included ALFRED genes (13 ALFRED genes, 3 known CPGs in ALFRED genes, 10 ALFRED genes without CPGs), the genes were added sequentially according to their ALFRED *P*-values for each cancer type, most significant gene (lowest *P*-value) first. For the four remaining gene sets that did not use information from the ALFRED test (i.e. ALFRED *P*-values), the genes were randomly ordered. For the full set of known CPGs, we first added genes known to predispose to each particular cancer type (e.g., *BRCA1* in ovarian cancer was added before others), after that randomly introducing the rest of the genes.

### Known cancer gene sets

A total of 110 known germline CPGs and the cancer types they predispose to were compiled from two sources; 67 from the Cancer Gene Census (CGC)^10^ and 99 from a recently published review paper including four Lynch syndrome-associated genes (*MLH1*, *MSH2*, *MSH6*, and *PMS2*)^2^ (Supplementary Data 14). Fifty-eight genes were shared between the two sets. In addition, 112 DNA damage response genes were obtained from a recently published review paper (37 genes overlapped with known CPGs)^37^. A total of 1695 somatic drivers were compiled from four sources: 409 known somatic cancer genes were from the CGC, 480 genes were compiled from nine sources including large-scale cancer studies, publicly available screening panels and unpublished analysis of public available data sets^38^, 876 candidate cancer genes from Broad Firehose determined by MutSig2CV^39^ (FDR ≤ 25% in any cancer type, http://gdac.broadinstitute.org/), and 431 candidate TSs and OGs by TUSON (FDR ≤ 20%)^40^. Three hundred and sixty-three compiled TS genes were obtained from Srivas et al.^41^, including CGC, driver genes by Vogelstein et al.^42^, CancerGenes resource by MSKCC^43^, and those predicted by TUSON. 209 OGs were obtained from CGC, from Vogelstein et al.^42^ and predicted by TUSON. With respect to overlap, 373 genes were common to at least two of the four sources and 1322 genes were present in only one data set. Within our 2983 tested genes, 46 were CPGs and 329 were somatic drivers (without overlap with CPGs) (Fig. 2a).

### Genomic data from TCGA

Data were obtained from TCGA Genome Data Analysis Center (GDAC) Firehose (http://gdac.broadinstitute.org/; downloaded in January 2016). Somatic variants were extracted from the level 4 Mutation Annotation Format (MAF) files in 8715 samples from 27 cancer types (8156 samples overlapped our set) and filtered to include only nonsynonymous variants in the coding region. Genomic copy number alteration (CNA) data (Affymetrix SNP6 platform) were extracted from the GISTIC2 (ref. ^44^) processing pipeline in 10,638 samples in 29 cancer types (9672 samples overlapped our set).

### Associating AI frequency with biological features

Estimated ploidies were derived from genome-wide copy number data using ABSOLUTE method in 4957 TCGA samples^45^, of which 4113 samples overlapped with our data (downloaded from https://www.synapse.org/#!Synapse:syn1710466). Sample purity was calculated from the ESTIMATE method by measuring noncancerous components of the tumor samples as reported in Aran et al.^46^ in 9364 TCGA samples (8276 samples overlapped with our data). The degree of intra-tumor heterogeneity (ITH) across 17 cancer types (11 cancer types were overlapped with our data) was obtained from McGranahan et al.^47^. It was defined as the absolute numbers of heterogeneous non-silent mutations divided by the sum of absolute numbers of heterogeneous and homogeneous non-silent mutations.

### Code availability

The LOH and randomization codes are available upon request.

### Data availability

This paper reanalyzes TCGA whole exome sequencing (retreived from https://cghub.ucsc.edu/) and control samples (WHI, https://esp.gs.washington.edu/drupal/; UK10K, http://www.uk10k.org/; 1000 genomes, http://www.internationalgenome.org/). TCGA and control data sets are available upon request from dbGaP under accession phs000178 (TCGA) and phs000200 (WHI), and the study authors (1000 genomes and UK10K). The data set of rare variants is from the ExAC Browser version 0.3(http://exac.broadinstitute.org/). All other relevant data are available from the corresponding author on request.

## Electronic supplementary material

Supplementary Information

Description of Additional Supplementary Files

Supplementary Data 1

Supplementary Data 2

Supplementary Data 3

Supplementary Data 4

Supplementary Data 5

Supplementary Data 6

Supplementary Data 7

Supplementary Data 8

Supplementary Data 9

Supplementary Data 10

Supplementary Data 11

Supplementary Data 12

Supplementary Data 13

Supplementary Data 14
