# Supplementary material for: Systematic discovery of germline cancer predisposition genes through the identification of somatic second hits
Source: Nat Commun. 2018 Jul 4;9:2601. doi: 10.1038/s41467-018-04900-7 (PMC6031629; doi:10.1038/s41467-018-04900-7)
Supplement: Supplementary file 1 — Supplementary Information [file 41467_2018_4900_MOESM1_ESM.pdf]

Supplementary information for:

Systematic discovery of germline cancer predisposition genes through the identification of somatic second hits

Park *et al*.

Includes: Supplementary Figures 1-12
Supplementary Table 1

Supplementary Figures

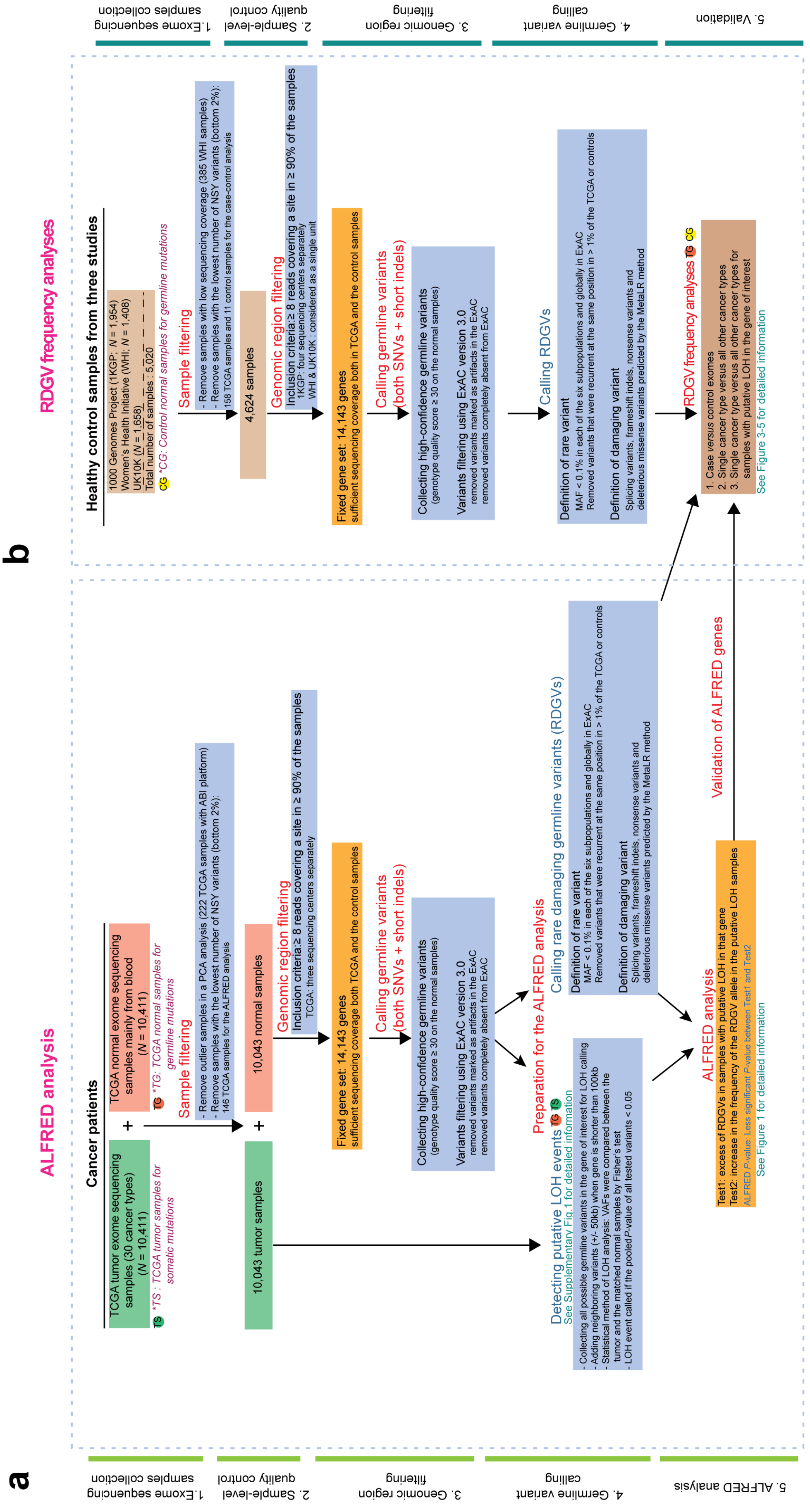

Supplementary Figure 1: Overall design for **a**, the ALFRED analysis using TCGA normal and tumor samples and **b**, Three validation analyses for ALFRED genes using TCGA normal samples and control samples from three studies (1000 Genomes Project, WHI and UK10K).

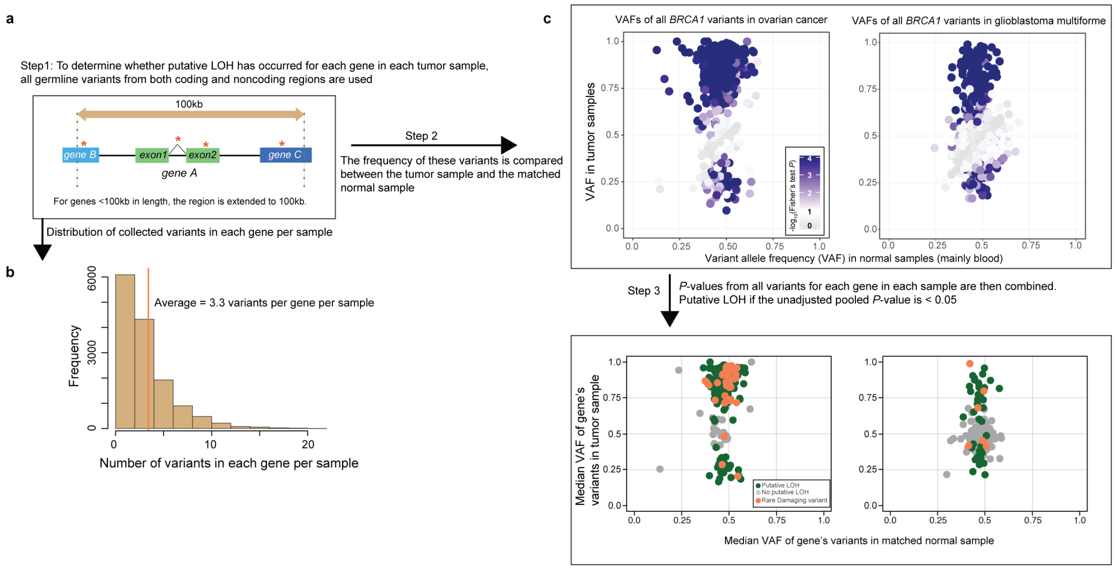

Supplementary Figure 2: Method for calling putative LOH events from cancer exome data, based on allelic imbalance (AI). **a**, To detect AI events in each gene, all coding and non-coding variants in a gene or within a 100kb window centered on the gene (when the gene is less than 100kb in length) are used. **b**, The distribution of the number of collected variants per gene per sample. **c**, Variant allele frequencies were compared between the tumor and the matched normal sample from the same individual by a Fisher's exact test on read counts (left panel). The *P*-values from all tested variants were then pooled for each gene and an AI event called if the pooled *P*-value was <0.05.

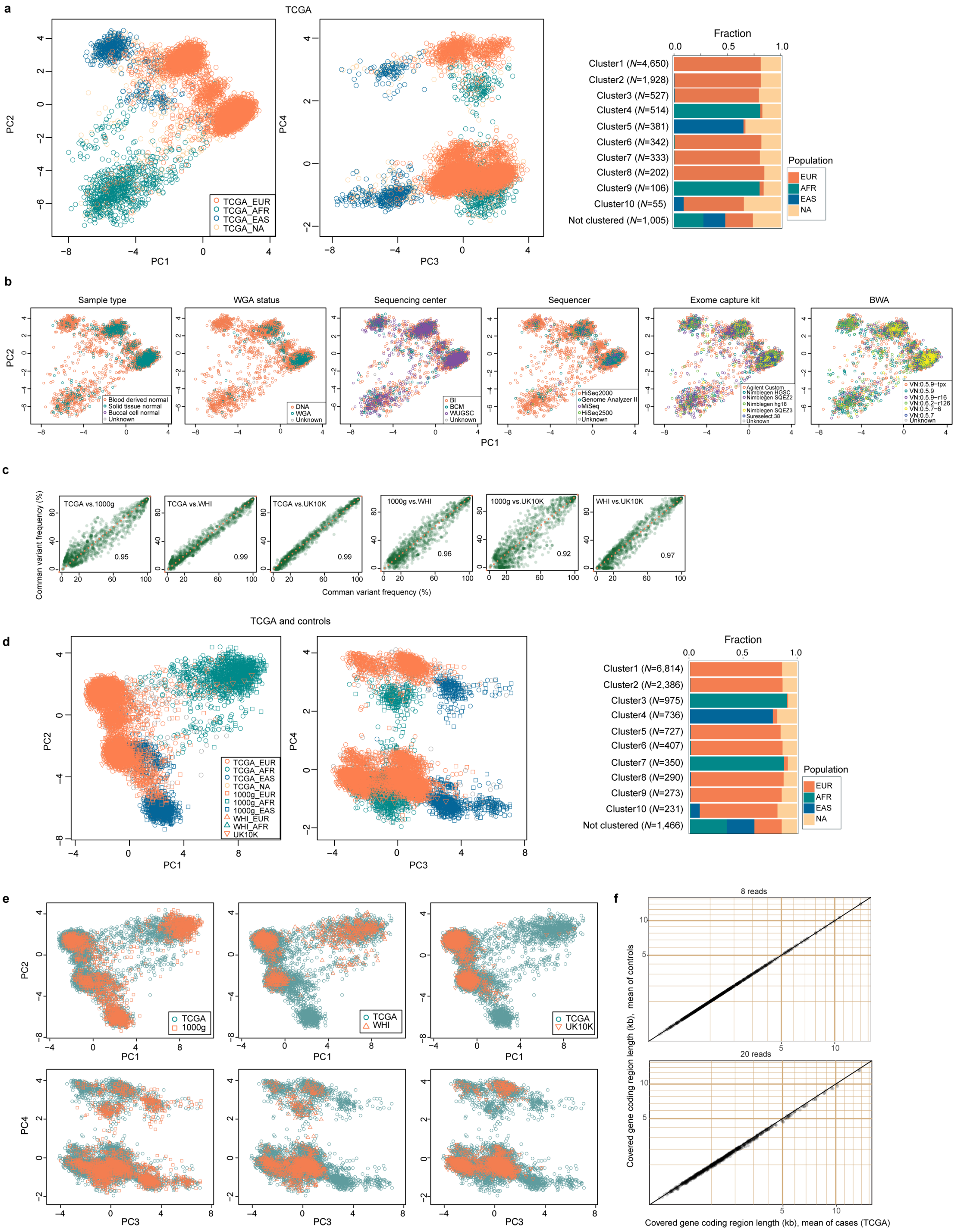

Supplementary Figure 3: Principal components analysis (PCA) of germline variants in TCGA and control samples reflects population structure. **a**, PCA was performed using common variants (population allele frequency > 5%) in the TCGA samples (cancer patients) to cluster them by their genetic relatedness. Samples are colored by the self-reported ethnicities of patients. Samples were clustered into ten clusters using PCs 1 to 4 and the samples that could not be assigned to a cluster were excluded from further analysis (See **Fig. 1**). **b**, PCA plots using TCGA samples were colored six technical covariates of TCGA samples. **c**, Pairwise comparisons of common variant frequencies across TCGA and three control exome datasets. The Pearson correlation coefficient is shown in each plot. **d**, PCA

performed using common variants in the TCGA samples together with control samples (general population). **e**, Data as in **d**, but shown for each control set separately. **f,** Comparison of sequencing coverage between TCGA samples (*N* = 10,031) and control samples (*N* = 4,624). The scatter plot shows the mean size (in kb) covered by at least 8 or 20 reads for all known CPGs (*N* = 109) and somatic drivers (*N* = 1,695) in the TCGA samples (x -axis) and in the control samples (y-axis).

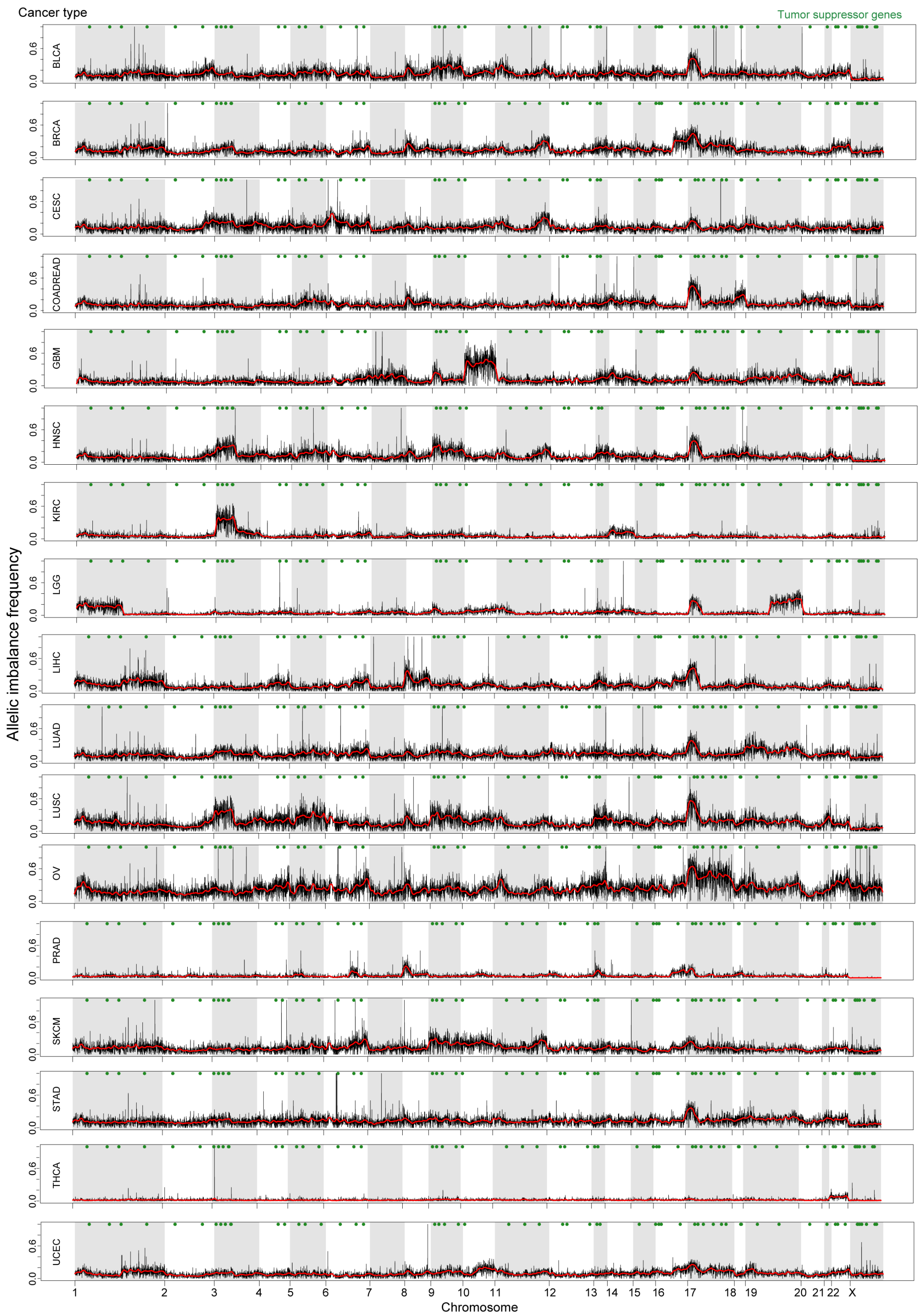

Supplementary Figure 4: Allelic imbalance landscape in the TCGA across 17 cancer types. The frequencies of putative LOH events (quantified as allelic imbalance) for the 14,143 examined genes are presented in black. Red line is a smoothed fit. Known tumor suppressors (*N* = 363; green circles; top) are displayed in their corresponding genomic position.

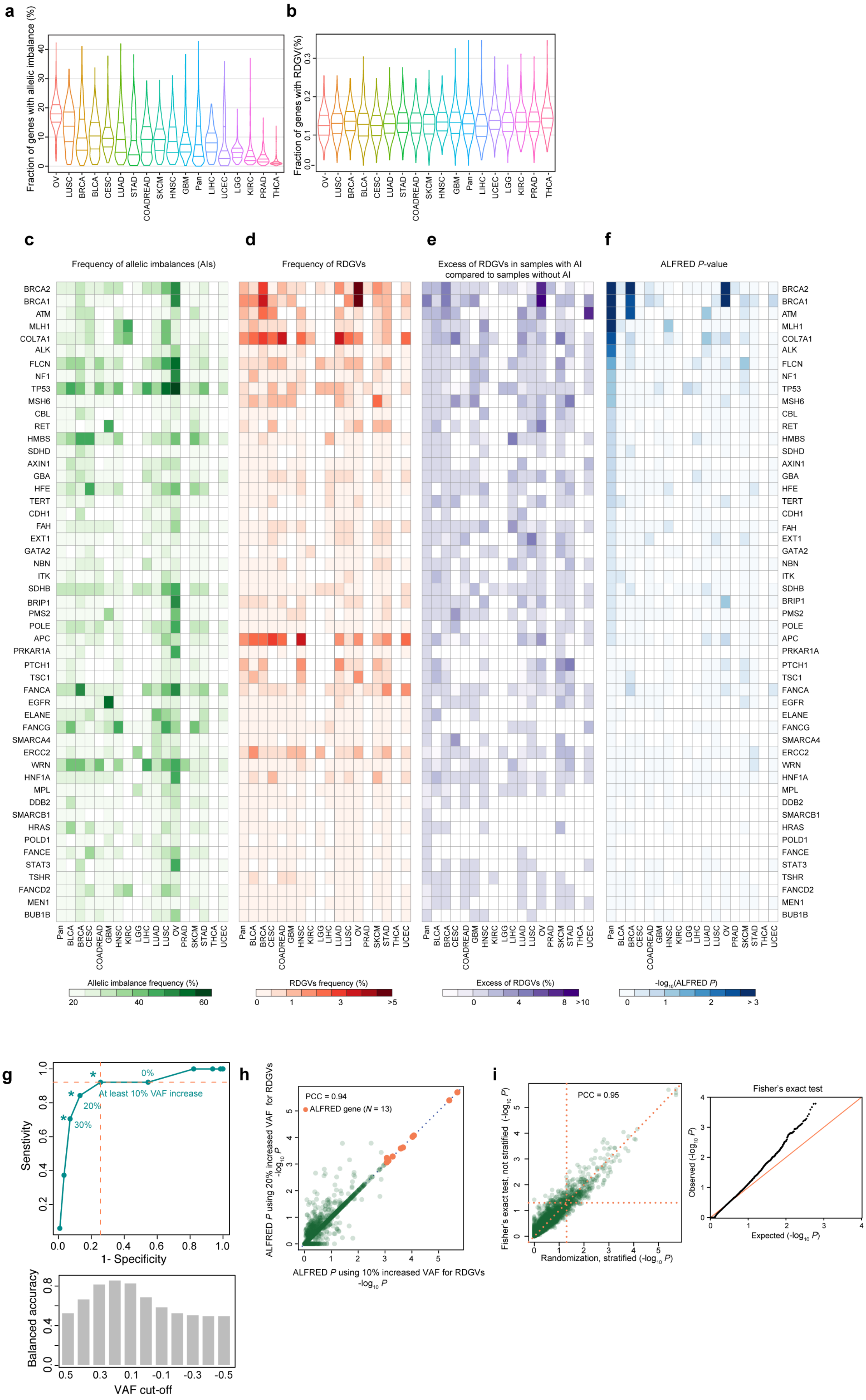
a
Fraction of genes with allelic imbalance (%)
OV LUSC BRCA BLCA CESC LUAD STAD COADREAD SKCM HNSC GBM Pan LIHC UCEC LGG KIRC PRAD THCA
b
Fraction of genes with RDGV(%)
OV LUSC BRCA BLCA CESC LUAD STAD COADREAD SKCM HNSC GBM Pan LIHC UCEC LGG KIRC PRAD THCA
c
Frequency of allelic imbalances (AIs)
d
Frequency of RDGVs
e
Excess of RDGVs in samples with AI compared to samples without AI
f
ALFRED P-value
BRCA2 BRCA1 ATM MLH1 COL7A1 ALK FLCN NF1 TP53 MSH6 CBL RET HMBS SDHD AXIN1 GBA HFE TERT CDH1 FAH EXT1 GATA2 NBN ITK SDHB BRIP1 PMS2 POLE APC PRKAR1A PTCH1 TSC1 FANCA EGFR ELANE FANCG SMARCA4 ERCC2 WRN HNF1A MPL DDB2 SMARCB1 HRAS POLD1 FANCE STAT3 TSHR FANCD2 MEN1 BUB1B
Pan BLCA BRCA CESC COADREAD GBM HNSC KIRC LGG LIHC LUAD LUSC OV PRAD SKCM STAD THCA UCEC
Allelic imbalance frequency (%)
20 40 60
RDGVs frequency (%)
0 1 3 >5
Excess of RDGVs (%)
0 4 8 >10
-log10(ALFRED P)
0 1 2 > 3
g
Senstivity
1- Specificity
At least 10% VAF increase
0%
20%
30%
Balanced accuracy
VAF cut-off
0.5 0.3 0.1 -0.1 -0.3 -0.5
h
PCC = 0.94
ALFRED gene (N = 13)
ALFRED P using 20% increased VAF for RDGVs -log10 P
ALFRED P using 10% increased VAF for RDGVs -log10 P
i
PCC = 0.95
Fisher's exact test, not stratified (-log10 P)
Randomization, stratified (-log10 P)
Fisher's exact test
Observed (-log10 P)
Expected (-log10 P)

Supplementary Figure 5: Distributions of genes with allelic imbalance (AI) and RDGVs in each cancer type. **a**, Distribution of AI frequencies, estimated across the 14,143 tested genes for each tumor sample, shown separately for cancer types. A value of 100% would indicate that AI was detected for all tested genes in a sample. The horizontal lines in each violin plot show the upper and lower quartiles and the median. **b**, Same as **a**, but showing frequencies of RDGVs in genes. 100% would indicate that all examined genes have RDGVs within a sample. The horizontal lines in each violin plot show the upper and lower quartiles, as well as the median. Heat map of **c**, AI frequency, **d**, RDGV frequency, **e**, Excess of RDGVs in samples with AI compared to samples without AI, and **f**, ALFRED *P*-values for known CPGs. All panels **c**-**f** show data for the pan-cancer analysis and for 17 individual cancer types. **g**, Sensitivity and specificity estimates for different thresholds of required VAF increase to call putative LOH events in ALFRED test 2 (see **Methods**). Rare truncation variants of six genes (*BRCA1*, *BRCA2*, *MSH6*, *PALB2*, *RAD51* and *TP53)* in ovarian cancer (*N*=51) were used as a true positive set, while randomly shuffled (100,000 times) VAFs between the tumors and matched normal samples were used as a true negative set. **h,** ALFRED *P* value ($-\log_{10} P$) comparisons between 10% VAF increase threshold, as used in ALFRED by default, and an alternative 20% VAF increase threshold. ALFRED genes are colored in red. **i**, A comparison between our stratified randomization method that accounts for population structure (left panel, x-axis) and performing a simple Fisher's exact test (one-sided) in a pan-cancer dataset (y-axis). Quantile-quantile (Q-Q) plot suggests inflated *P* values would be obtained if a simple Fisher's test were used on this data set.

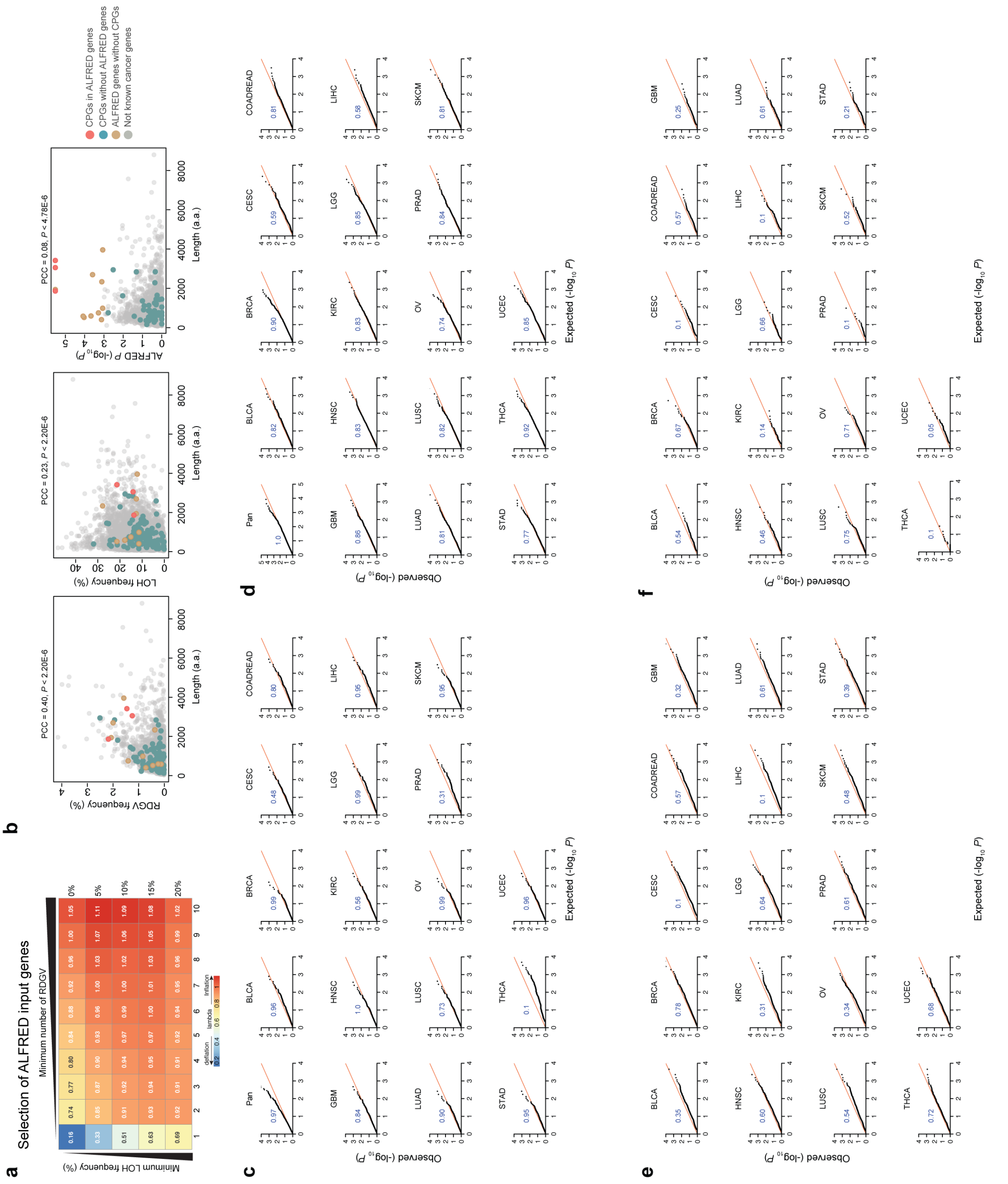

Supplementary Figure 6: *P*-value distribution from the ALFRED analyses and three RDGV frequency analyses. **a**, Heat map of lambda values from the ALFRED analyses in a pan-cancer analysis by changing the threshold of requiring LOH frequency and RDGV frequency. Quantile-quantile (Q-Q) plots of *P*-values from the **b**, Comparison between length and RDGV frequency, LOH frequency and ALFRED *P*-value (as $-\log_{10} P$). The Pearson correlation coefficient (PCC) is shown. **c**, ALFRED analyses (enrichment of RDGVs in allelic imbalance (AI) tumor samples compared with non-AI tumor samples), **d**, Case-control analyses (enrichment of RDGVs in cancer patients compared with control samples), **e**, Enrichment of RDGVs in one cancer type compared to in all the other cancer types, **f**, Enrichment of RDGVs in one cancer type compared to in all the other cancer types, restricted to putative LOH

samples (estimated via AI). The values of the inflation factor λ are shown on the plots; λ ≤ 1.0 indicates no inflation.

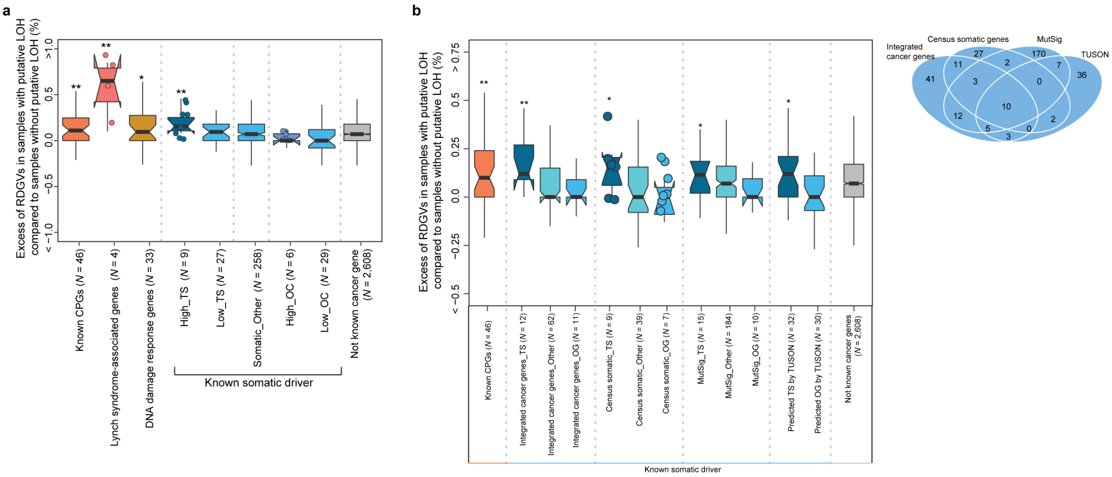

Supplementary Figure 7: Excess of RDGVs in tumor samples with putative LOH events (estimated via allelic imbalance, AI) over samples without putative LOH. **a**, Shown for Lynch syndrome associated genes (*MLH1*, *MSH2*, *MSH6* and *PMS2*), DNA damage repair-associated genes, high confidence somatic driver genes (reported in at least three out of four data sources, "High") or low confidence somatic drivers (one or two data sources, "Low"). **b**, Shown for individual data sources. Known CPGs do not overlap with somatic drivers, as shown in **Fig. 2**, but the four sets of somatic drivers overlap each other as shown in the Venn diagram (* $P < 5.0 \times 10^{-2}$, ** $P < 5.0 \times 10^{-3}$). The median value of each gene set is displayed as a band inside each box. The length of each whisker is 1.5 times the interquartile range (shown as the height of each box).

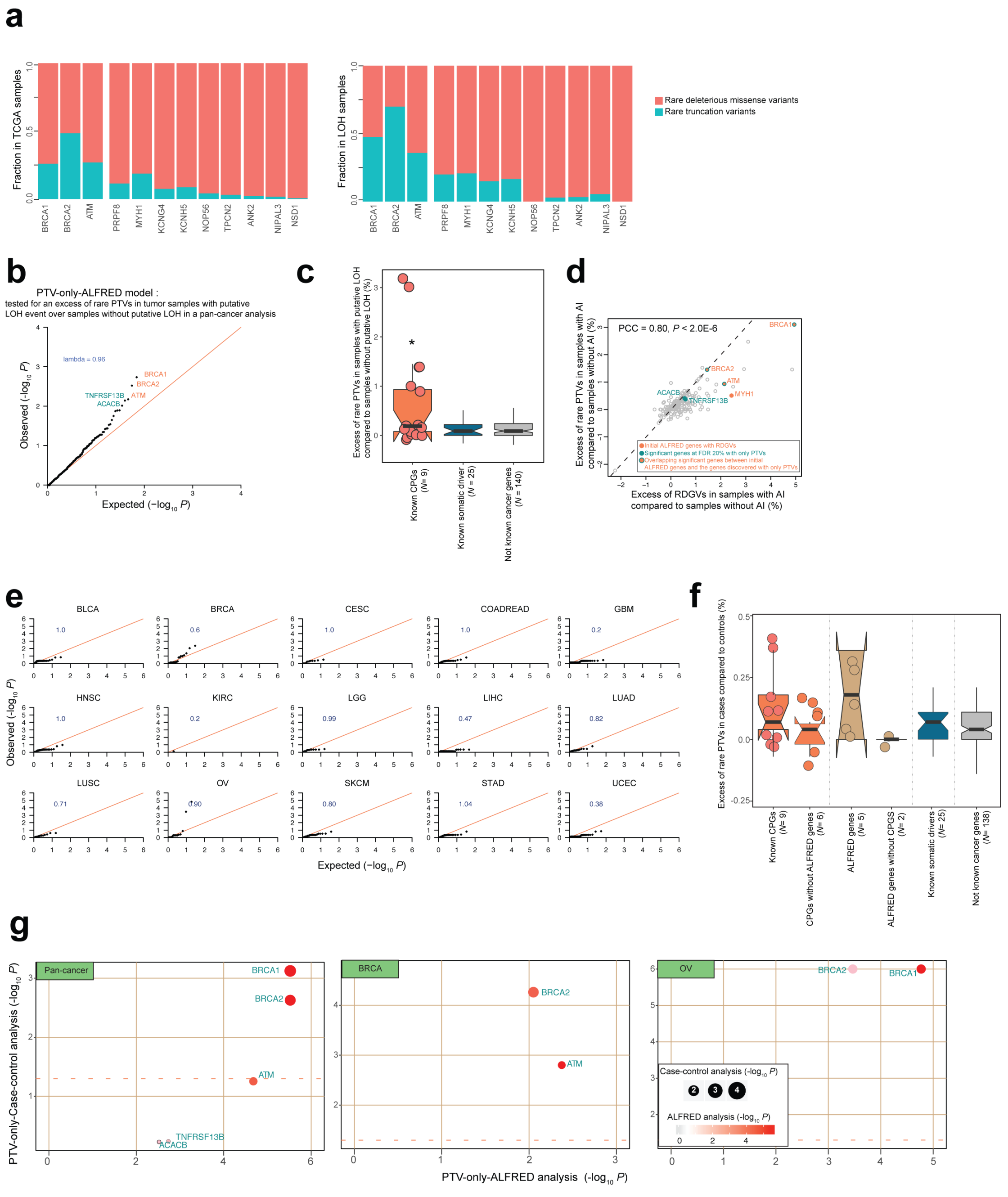

Supplementary Figure 8: Excess of rare protein truncation variants (PTVs) in AI samples. **a**, Fraction of rare truncation variants (splicing variants, frame-shift indels, nonsense variants) from RDGV in ALFRED genes. **b**, Q-Q plot of *P*-values from the PTV-only-ALFRED model (enrichment of rare-PTVs in samples with AI to samples without AI) in a pan-cancer analysis. Detected genes at FDR = 20% are labeled, of which three genes (*BRCA1/2* and *ATM;* red color) overlap with ALFRED initial design (RDGV excess), while *TNFRSF13B* and *ACACB* (blue color) are newly detected. **c**, Enrichment of rare PTVs in samples with allelic imbalance over samples without allelic imbalance for different gene sets (* $P < 5.0 \times 10^{-2}$). The median value of each gene set is displayed as a band inside each box. The length of each whisker is 1.5 times the interquartile range (shown as the height of each box). All values lying outside the whiskers are considered to be outliers. **d**, ALFRED effect size comparison between initial

ALFRED model (x-axis; excess of RDGVs in samples with AI over samples without AI) *versus* PTV-only-ALFRED model (y-axis; excess of rare PTVs in samples with AI over samples without AI). The Pearson correlation coefficient (PCC) is shown. The black line corresponds to slope = 1. **e**, Q-Q plot of *P*-values from the PTV-only-ALFRED model (enrichment of rare PTVs in samples with AI to samples without AI) in 15 cancer types. Two cancer types (PRAD and THCA) were not presented due to the number of tested genes being less than five. Values of the inflation factor λ are shown on the plots. **f**, Enrichment of rare PTVs in 10,031 cancer cases over 4,624 controls for six gene sets. **g**, Case-control analyses with only rare PTVs for the pan-cancer and two individual cancer types.

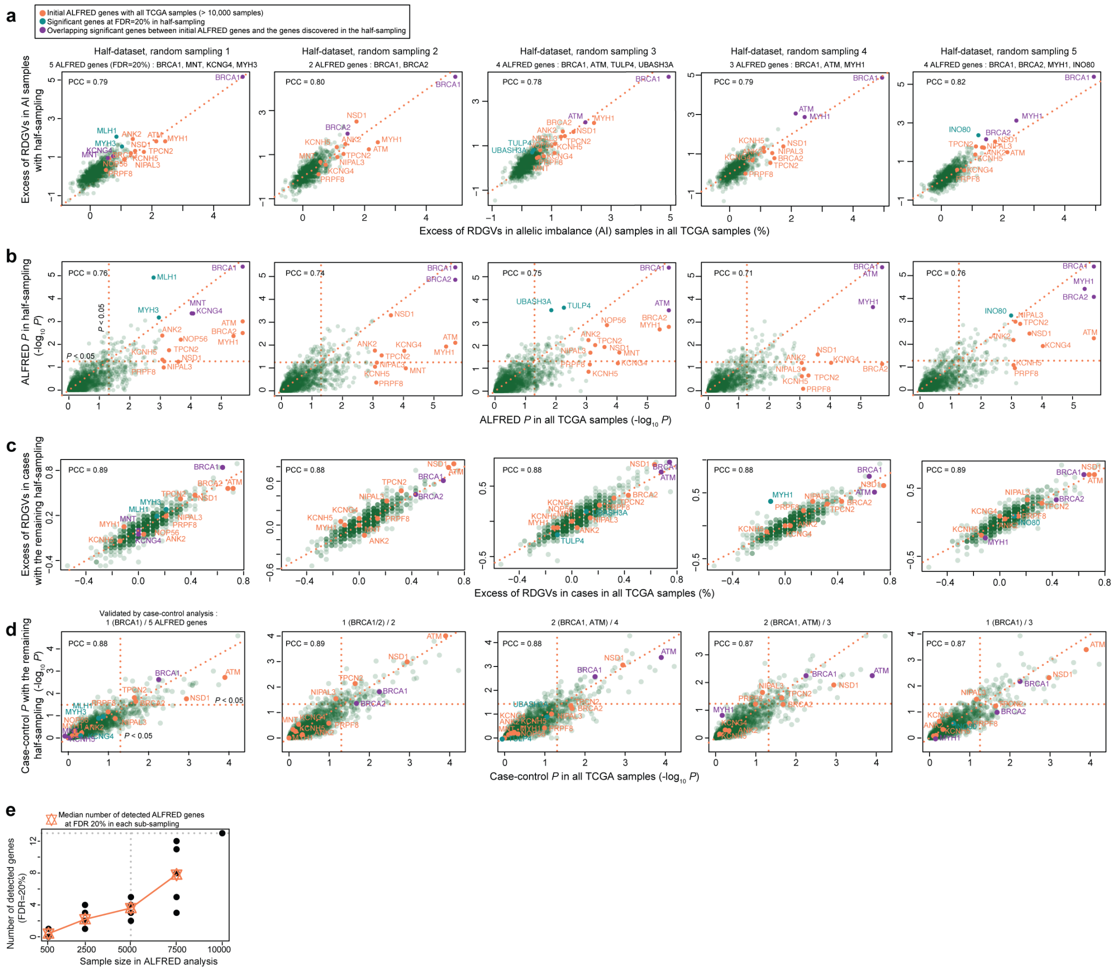

Supplementary Figure 9: Sub-sampling analysis. TCGA samples were randomly split in two groups, using the first half of the data for the ALFRED analysis and the second half for the case-control analysis. **a**, Comparison between the initial ALFRED effect size (excess of RDGVs in all TCGA samples with AI to samples without AI) *versus* five half-samplings. The Pearson correlation coefficient (PCC) is shown. The red line corresponds to slope = 1. **b**, Comparison between initial ALFRED *P*-values (using > 10,000 tumor samples) to ALFRED *P*-values of five half-samplings. **c**, Comparison between initial effect sizes in the case-control analysis (excess of RDGVs in all TCGA samples compared to control samples) *versus* effect size in the second half of TCGA samples. **d**, Comparison between initial case-control *P*-values in all TCGA samples *versus* case-control *P*-values in the remaining half of the TCGA samples. **e**, Saturation analysis by sub-sampling tumor samples from the pan-cancer data set suggests that the number of detected genes is not saturated. Each point indicates randomly selected sub-samples from > 10,000 TCGA samples and the red dot indicates the median number of detected ALFRED genes at FDR 20% across the five simulation runs.

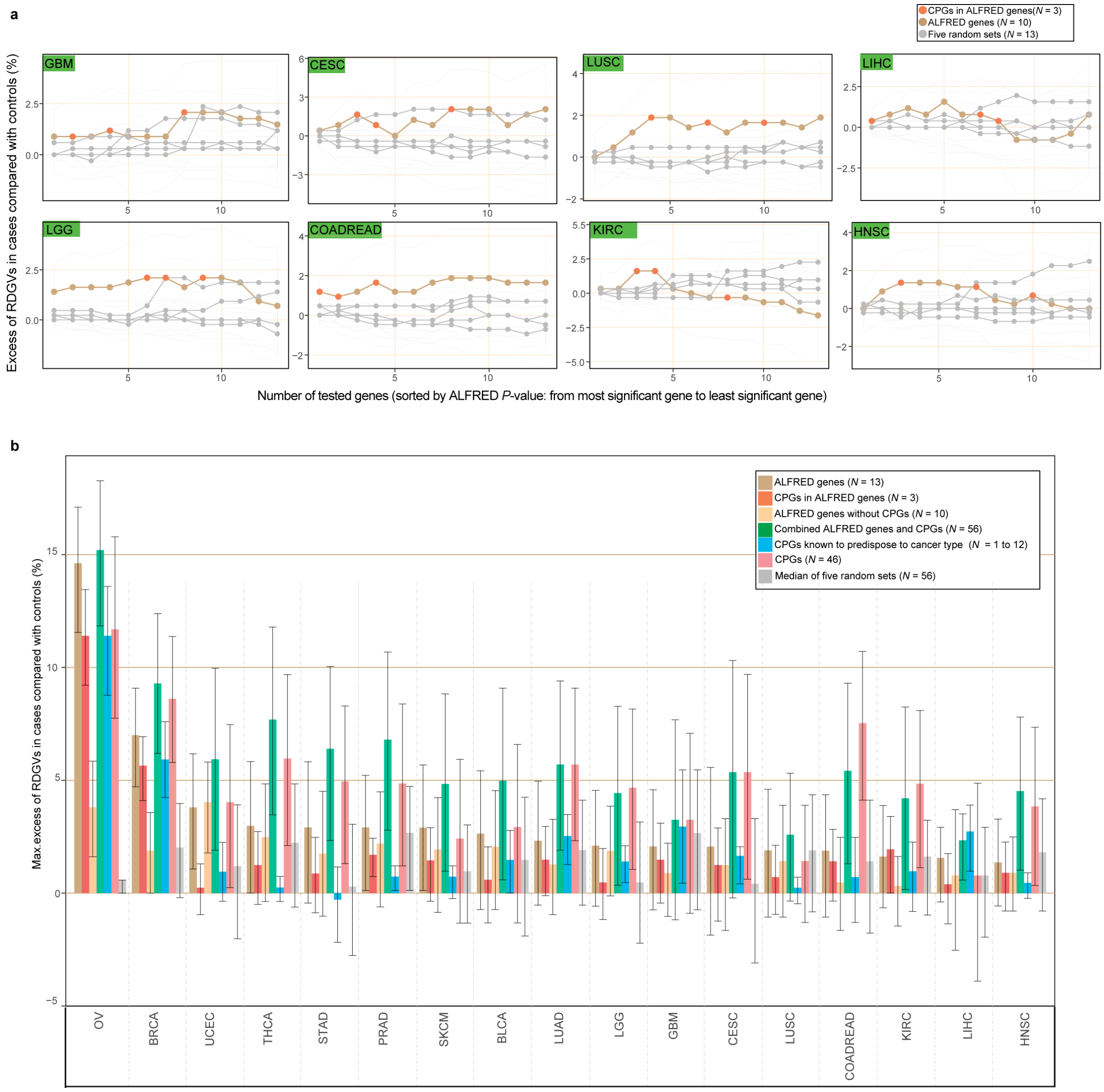

Supplementary Figure 10: Contribution of ALFRED genes to cancer risk. **a**, Maximum excess of RDGVs in cases (TCGA cancer patients) compared to controls (general population), measured by adding ALFRED genes sequentially from most significant gene (left) to least significant gene (right), ordered by their ALFRED *P*-value. Genes were added in a random order in the random gene sets (grey lines). **b**, Comparison of maximum excess of RDGVs in cases compared to controls using seven gene sets: ALFRED genes, ALFRED genes overlapping known CPGs, ALFRED genes excluding known CPGs, combination of ALFRED genes and known CPGs, CPGs known to predispose to particular cancer types, CPGs as a full set, and random genes. For the random gene set, we randomly selected the same number of genes as in the largest gene set – the combined ALFRED genes and CPGs (N = 56) – five times and used the median value of their excesses. Genes were randomly added in the four gene sets that did not use information from the ALFRED *P*-values. Error bars indicate 95% confidence interval of the excess, obtained via randomization.

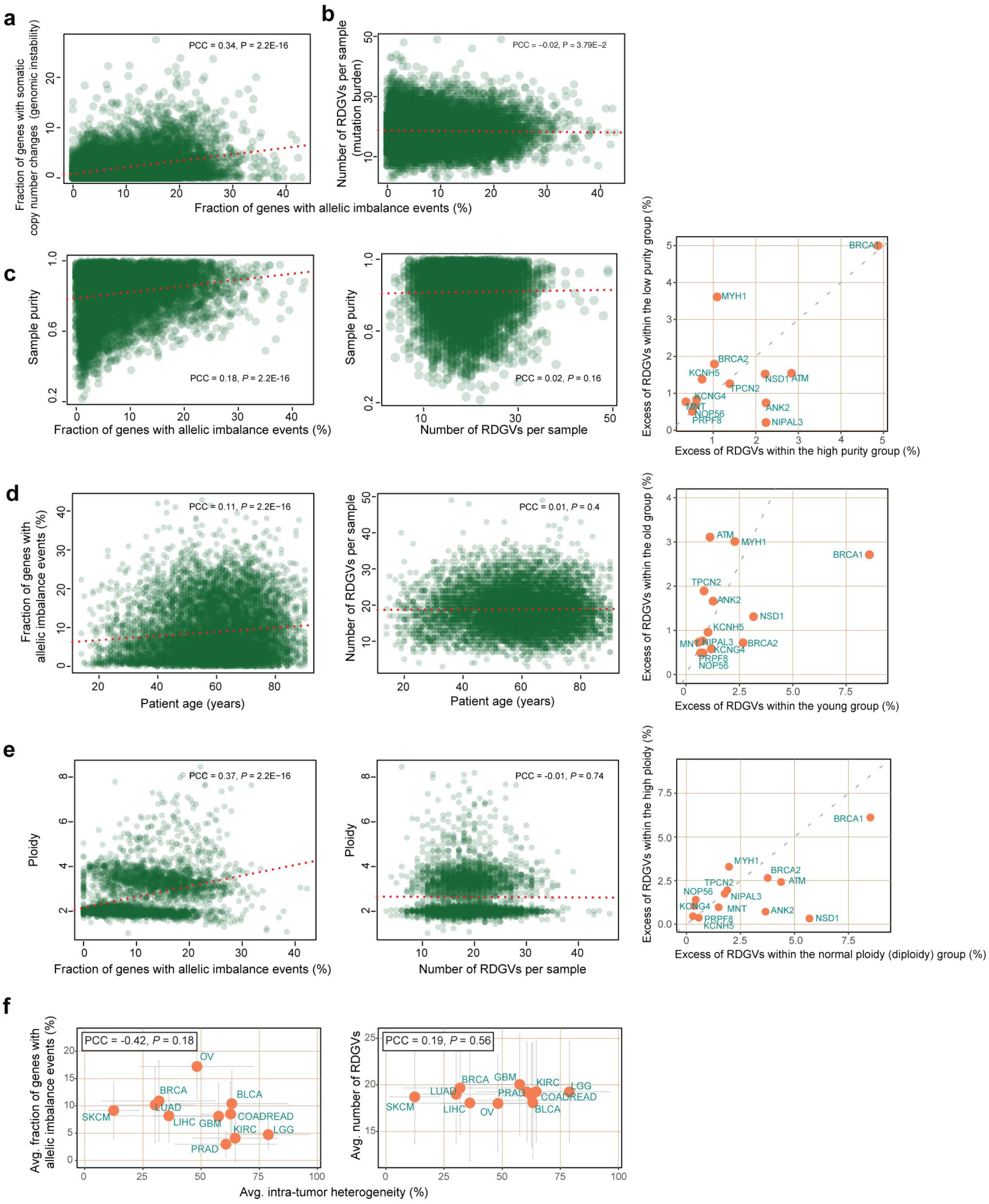

Supplementary Figure 11: Relationship between allelic imbalance (AI) frequency and biological features of tumor samples. **a**, Each point shows fraction of genes with somatic copy number changes (amplification or deletion; genomic instability) *versus* fraction of genes with AI events in the pan-cancer data set. **b**, Each point shows the number of RDGVs per sample (mutation burden) *versus* fraction of genes with AI events in the pan-cancer data. The Pearson correlation coefficient (PCC) and its *P*-value are shown. Red line is a linear fit. **c**, Relationship of sample purity with (i) fraction of genes with AI events in 8,276 TCGA samples (left panel) and (ii) mutation burden of RDGVs (middle panel) in the pan-cancer data. Sample purity was obtained from Aaran *et al.*[1] Right panel shows the ALFRED *P*-value comparison ($-\log_{10} P$) between a high purity group (sample purity above median purity across all samples)

and in a low purity group, shown for ALFRED genes (*N* = 13). **d**, Relationships of patient age with fraction of genes with AI events (left panel) and with mutation burden of RDGVs (middle panel). ALFRED *P*-value comparison between young patients (age < 60) and older patients (right panel). **e**, Relationship of ploidy with: AI frequency (left panel) and with RDGV burden (middle panel) in 4,113 TCGA samples. Ploidy was estimated by ABSOLUTE[2]. ALFRED *P*-value comparison a high ploidy group (ploidy > 2.5) and a normal ploidy (diploid) group (right panel). **f**, Scatter plot of the average intra-tumor heterogeneity (ITH) from McGranahan *et al.* [3] *versus* (i) average fraction of genes with AI events (left panel) and (ii) average number of RDGVs (right panel) for each of the 11 cancer types with ITH data available. ITH was defined as the absolute numbers of heterogeneous non-silent mutations divided by the sum of absolute numbers of heterogeneous and homogeneous non-silent mutations [3]. Error bars represent the standard deviation.

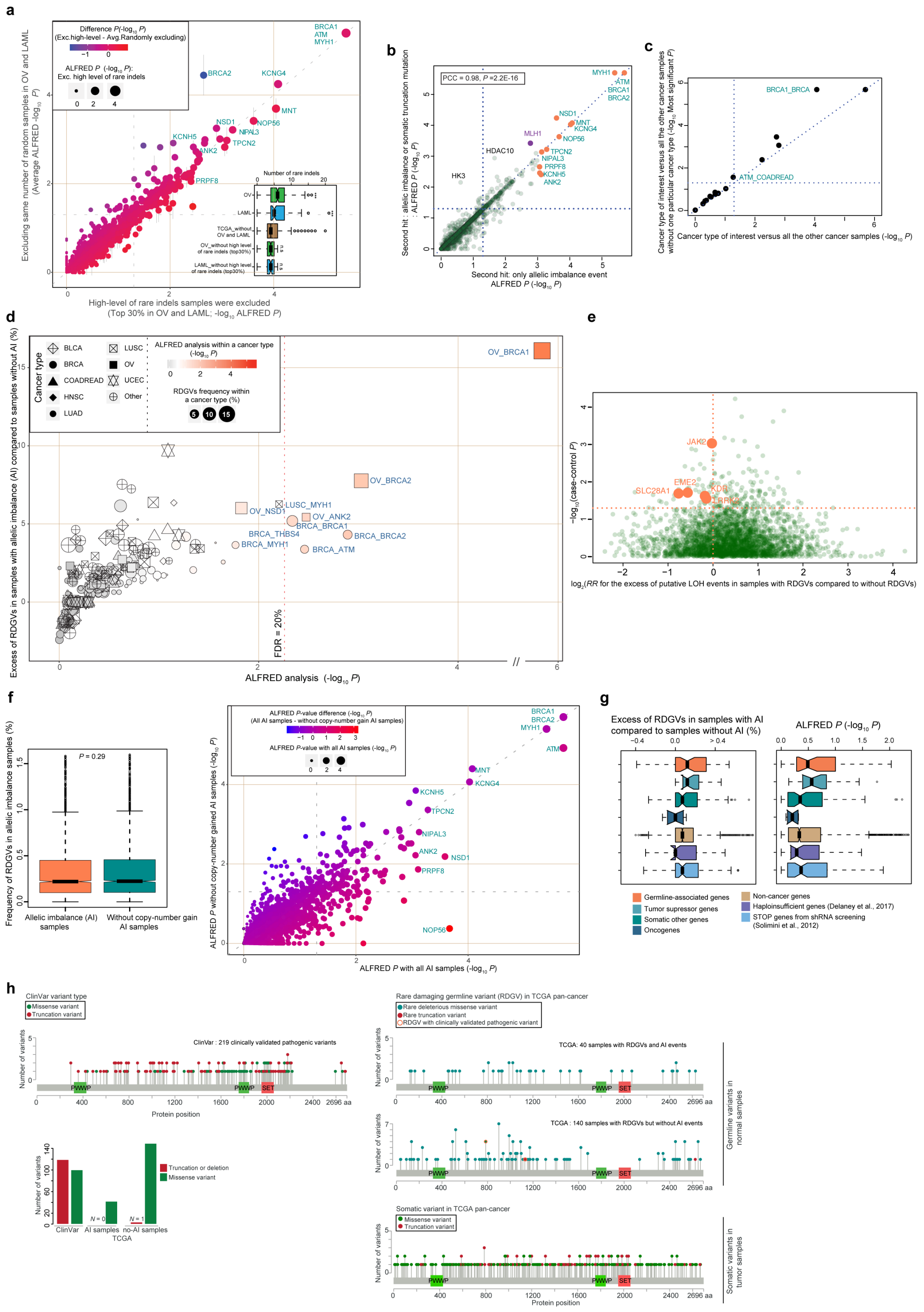

Supplementary Figure 12: Robustness of the ALFRED method. **a**, ALFRED *P*-values after removing OV and LAML samples with high rare indel (MAF < 0.1%) counts (top 30%; x-axis) and after randomly excluding the same number of samples in OV and LAML (y-axis), repeated three times. ALFRED genes are labeled (*N* = 13). Error bars represent s.d. Box plot shows the distribution of the number of rare indels in the OV and LAML data sets. **b**, ALFRED *P*-value comparison ($-\log_{10} P$) between the approach where only AI events (putative

LOH) are considered as a second hit (x-axis; the default setting in the ALFRED method) *versus* the approach where either AI events or somatic truncation mutation are considered as a second hit (y-axis). ALFRED genes are labeled, and additionally *MLH1* which is significant on the y-axis only. The Pearson correlation coefficient and its *P*-value are shown. **c**, Significance of RDGVs in ALFRED genes in each cancer type compared to in all other cancer types but excluding one of the remaining types, which is repeated for all 'other' cancer types one-by-one (16 times). **d**, Excess of RDGVs in samples with AI are plotted against the ALFRED randomization test across 17 cancer types. FDR correction was done across 17 cancer types pooled together. Color indicates significance and shape indicates cancer type. **e**, Relative risk (RR) for the excess of putative LOH events in samples with RDGVs compared to without RDGVs (x-axis) *versus* excess of RDGVs in cases over controls (y-axis) for the pan-cancer data. **f**, ALFRED *P*-value comparison ($-\log_{10} P$) between using all AI samples as putative LOH events *versus* after excluding copy-number gained AI samples and considering them as a 'non-LOH group'. **g**, Enrichment of RDGVs in samples with AI over samples without AI for haploinsufficient genes[4] and 'STOP' genes which negatively regulated proliferation from shRNA screening from Solimini *et al* [5]. The median value of each gene set is displayed as a band inside each box. The length of each whisker is 1.5 times the interquartile range (defined as the height of each box). All values lying outside the whiskers are considered to be outliers. **h**, Distribution of *NSD1* variants causing Sotos syndrome (left panel) contrasted to rare damaging germline variants or somatic variants in *NSD1* in TCGA cancer patients (right panel). Clinically-validated pathogenic variants were obtained from ClinVar [6].

Supplementary Table 1. Pan-cancer case-control analysis for the ALFRED genes.

Gene	Significantly enriched cancer type by ALFRED (FDR 20%)	Number of samples with RDGVs in case samples	Number of samples with RDGVs in control samples	*P*-value	Excess of RDGVs in case samples compared with control samples (%)	Lower_95% CI	Upper_95% CI
ATM	Pan, BRCA	114	20	1.24E-04	0.68	0.3	1.04
BRCA1	Pan, BRCA, OV	194	57	5.60E-03	0.64	0.16	1.15
BRCA2	Pan, BRCA, OV	132	40	2.09E-02	0.43	0.02	0.84
MYH1	Pan	198	94	6.86E-01	-0.11	-0.64	0.43
KCNG4	Pan	17	9	5.76E-01	0	-0.16	0.18
MNT	Pan	0	1	1.00E+00	0	-0.04	0
NOP56	Pan	24	9	5.95E-01	0	-0.18	0.18
NSD1	Pan	179	48	1.14E-03	0.72	0.27	1.2
TPCN2	Pan	82	20	2.26E-02	0.32	0.02	0.66
ANK2	Pan, OV	138	57	4.65E-01	0.04	-0.38	0.48
KCNH5	Pan	74	39	7.96E-01	-0.14	-0.45	0.23
NIPAL3	Pan	59	19	6.80E-02	0.21	-0.05	0.52
PRPF8	Pan	34	8	1.02E-01	0.14	-0.05	0.36

Supplementary References

1 Aran, D., Sirota, M. & Butte, A. J. Corrigendum: Systematic pan-cancer analysis of tumour purity. *Nature communications* **7**, 10707, doi:10.1038/ncomms10707 (2016).

2 Carter, S. L. *et al.* Absolute quantification of somatic DNA alterations in human cancer. *Nature biotechnology* **30**, 413-421, doi:10.1038/nbt.2203 (2012).

3 McGranahan, N. & Swanton, C. Clonal Heterogeneity and Tumor Evolution: Past, Present, and the Future. *Cell* **168**, 613-628, doi:10.1016/j.cell.2017.01.018 (2017).

4 Delaney, J. R. *et al.* Haploinsufficiency networks identify targetable patterns of allelic deficiency in low mutation ovarian cancer. *Nature communications* **8**, 14423, doi:10.1038/ncomms14423 (2017).

5 Solimini, N. L. *et al.* Recurrent hemizygous deletions in cancers may optimize proliferative potential. *Science* **337**, 104-109, doi:10.1126/science.1219580 (2012).

6 Landrum, M. J. *et al.* ClinVar: public archive of relationships among sequence variation and human phenotype. *Nucleic acids research* **42**, D980-985, doi:10.1093/nar/gkt1113 (2014).
