# Supplementary material for: Systematic discovery of germline cancer predisposition genes through the identification of somatic second hits
Source: Nat Commun. 2018 Jul 4;9:2601. doi: 10.1038/s41467-018-04900-7 (PMC6031629; doi:10.1038/s41467-018-04900-7)
Supplement: Supplementary file 2 — Description of Additional Supplementary Files [file 41467_2018_4900_MOESM2_ESM.docx]

**Description of Additional Supplementary Files**

File Name: Supplementary Data 1

Description: Sample IDs and cancer types for the 10,043 TCGA cases included in this study.

File Name: Supplementary Data 2

Description: Frequencies (%) of putative LOH events (AIs) and RDGVs in all samples (pan-cancer and for each of the 17 cancer types separately).

File Name: Supplementary Data 3

Description: Pan-cancer ALFRED analysis.

File Name: Supplementary Data 4

Description: ALFRED analysis in 17 individual cancer types.

File Name: Supplementary Data 5

Description: ALFRED analysis in 17 individual cancer types for the ALFRED genes.

File Name: Supplementary Data 6

Description: Case-control analyses for 17 individual cancer types for the ALFRED genes.

File Name: Supplementary Data 7

Description: Case-control analyses for 17 individual cancer types for the ALFRED genes.

File Name: Supplementary Data 8

Description: Cancer type-specificity tests only using samples with AI (enrichment of RDGVs in one cancer type versus in all the other samples, only for AI samples) for the ALFRED genes.

File Name: Supplementary Data 9

Description: Summary of the ALFRED analyses and three RDGV frequency analyses for the ALFRED genes.

File Name: Supplementary Data 10

Description: ALFRED analysis in 17 individual cancer types when FDR correction was performed across 17 cancer types considered together.

File Name: Supplementary Data 11

Description: Pan-cancer ALFRED analysis with putative LOH-or-somatic truncating-mutation model.

File Name: Supplementary Data 12

Description: Pan-cancer rare PTV-only-ALFRED analysis.

File Name: Supplementary Data 13

Description: Rare PTV-only-ALFRED analysis in 17 individual cancer types.s

File Name: Supplementary Data 14

Description: Known cancer predisposition genes (CPGs) used in this study.
